# In Vivo Characterization of Endogenous Cardiovascular Extracellular Vesicles in Larval and Adult Zebrafish

**DOI:** 10.1161/ATVBAHA.121.316539

**Published:** 2021-07-15

**Authors:** Aaron Scott, Lorena Sueiro Ballesteros, Marston Bradshaw, Chisato Tsuji, Ann Power, James Lorriman, John Love, Danielle Paul, Andrew Herman, Costanza Emanueli, Rebecca J. Richardson

**Affiliations:** 1 School of Physiology, Pharmacology & Neuroscience, Faculty of Biomedical Sciences (A.S., M.B., C.T., J.L., D.P., R.J.R.); 2 Flow Cytometry Facility, Faculty of Biomedical Sciences (L.S.B., A.H.); 3 Bristol Heart Institute, School of Clinical Science (C.E.), University of Bristol, United Kingdom.; 4 Now with Charles River Laboratories, Discovery House, Quays Office Park, Conference Avenue, Portishead, Bristol, United Kingdom (L.S.B.).; 5 BioEconomy Centre, The Henry Wellcome Building for Biocatalysis, Biosciences, University of Exeter, United Kingdom (A.P., J.L.).; 6 National Heart and Lung Institute, Imperial College London, United Kingdom (C.E.).

**Keywords:** cardiovascular, exosomes, extracellular vesicle, flow cytometry, zebrafish

## Abstract

**Objective:**

Extracellular vesicles (EVs) facilitate molecular transport across extracellular space, allowing local and systemic signaling during homeostasis and in disease. Extensive studies have described functional roles for EV populations, including during cardiovascular disease, but the in vivo characterization of endogenously produced EVs is still in its infancy. Because of their genetic tractability and live imaging amenability, zebrafish represent an ideal but under-used model to investigate endogenous EVs. We aimed to establish a transgenic zebrafish model to allow the in vivo identification, tracking, and extraction of endogenous EVs produced by different cell types.

**Approach and Results:**

Using a membrane-tethered fluorophore reporter system, we show that EVs can be fluorescently labeled in larval and adult zebrafish and demonstrate that multiple cell types including endothelial cells and cardiomyocytes actively produce EVs in vivo. Cell-type specific EVs can be tracked by high spatiotemporal resolution light-sheet live imaging and modified flow cytometry methods allow these EVs to be further evaluated. Additionally, cryo electron microscopy reveals the full morphological diversity of larval and adult EVs. Importantly, we demonstrate the utility of this model by showing that different cell types exchange EVs in the adult heart and that ischemic injury models dynamically alter EV production.

**Conclusions:**

We describe a powerful in vivo zebrafish model for the investigation of endogenous EVs in all aspects of cardiovascular biology and pathology. A cell membrane fluorophore labeling approach allows cell-type specific tracing of EV origin without bias toward the expression of individual protein markers and will allow detailed future examination of their function.

## Abstract 2

Supplemental Digital Content is available in the text.

**Highlights**

- Multiple cell types produce extracellular vesicles of multiple morphologies in larval and adult zebrafish in vivo, including endothelial cells and cardiomyocytes.
- A transgenic membrane-tethered fluorophore system allows extracellular vesicles from specific cell types to be visualized, tracked, and obtained for downstream investigations.
- Extracellular vesicles are transferred between multiple different cell types in the adult zebrafish heart.
- The production of endogenous extracellular vesicles is altered following tissue injury.

Extracellular vesicles (EVs) are plasma membrane–bound particles produced and released by most cell types. EVs can be split into 3 classes: exosomes, formed from an endocytic pathway, microvesicles that are shed from the cell surface, and apoptotic bodies, which are shed from cells undergoing apoptosis.^1,2^ EVs can be trafficked locally and systemically and have been isolated from various biological fluids, including blood^3,4^ and pericardial fluid.^5,6^ Various surface glycans, lipids, and proteins reportedly guide EVs to regions of ECM (extracellular matrix) or recipient cells, and these interactions play integral roles in communicatory pathways.^7^ EVs play roles in homeostasis^8,9^ and are implicated in the progression of many diseases, including cardiovascular disease.^10,11^ EVs have been reported to facilitate communication between multiple cell types within the cardiac microenvironment, including cardiomyocytes (CMs), fibroblasts, immune and endothelial cells (ECs),^12–15^ and some populations of EVs are thought to be proangiogenic and cardioprotective.^11,15,16^

**See cover image**

Zebrafish have emerged as a powerful model for cell/developmental biology and human diseases and have many advantages including high fecundity, rapid external development, genetic tractability, and unrivaled cellular level in vivo imaging.^17^ Most zebrafish studies have been performed at larval stages; however, the advantages offered by adult zebrafish, including retained regenerative capacity, have led to an increasing number of studies using this model.^17^ Clinically relevant models of tissue injury that allow subsequent evaluations of regenerative processes are well established in adult zebrafish.^18^ In the heart, correct cardiac regeneration, including complete cardiomyocyte renewal, has been shown to be reliant on multiple different cell types including inflammatory cells and ECs,^19–22^ implying the need for a complex communication system. Little is known about the role of endogenous EVs in regenerative contexts despite the potential to identify proregenerative signals being exchanged between cell types.

The potential of EVs as biomarkers of disease and as novel therapeutic delivery vehicles has generated significant interest in recent years.^4,23^ However, the ability to reliably define the heterogeneous spectrum of endogenous EV subtypes and their functional significance in vivo is still in its infancy.^2^ The majority of EV characterization to date, by necessity, has been performed in vitro.^2^ Recent studies have developed novel ways to label exogenous EVs allowing their biodistribution and functional roles to be investigated in vitro and in vivo.^5,24–26^ Whereas these studies using exogenous EVs are beginning to identify important roles in multiple tissues and disease states, they are unable to address the full complexity of endogenous in vivo EV populations. Recent reports have started to bridge this gap by investigating endogenously produced EVs in larval zebrafish, including apoptotic bodies^27^ and CD63+ exosomes.^28^ However, in vivo studies of endogenous EVs released by ECs and CMs under homeostatic and disease conditions have not been attempted so far.

Here, we describe techniques using stable transgenesis to fluorescently label native cell-type specific EVs in vivo in larval and adult zebrafish, allowing these vesicles to be tracked, extracted, and validated. We demonstrate that global zebrafish EVs as well as EC- and CM-derived EVs can be observed in the peripheral circulation and the pericardial space, respectively. We report that by using adapted flow cytometry techniques these cell-type specific EVs can be analyzed and purified from tissue samples and evaluated ex vivo. Additionally, we describe total endogenous zebrafish EVs with multiple sizes and morphologies revealed by cryo electron microscopy (cryo-EM). Importantly, we describe the transfer of EVs between different cell types resident in the adult heart. Finally, we have used models of tissue injury in larval and adult zebrafish to demonstrate dynamic changes to the EV profile.

## Materials and Methods

The authors declare that all supporting data are available within the article (and its Data Supplement). Please also see the Major Resources Table in the Data Supplement.

### Zebrafish Lines and Procedures

The Tg(*actb2:HRAS-EGFP*),^29^ Tg(*tbp:GAL4*);(*UAS:secA5-YFP*),^30^ Tg(*kdrl:mCherry-CAAX*),^31^ Tg(*myl7:GFP*),^32^ Tg(*fli1:EGFP*),^33^ Tg(*mpeg1:EGFP*)gl22,^34^ and Tg(*myl7:HRAS-mCherry*)^35^ lines have been described previously. In all cases, animals were anesthetized via immersion in 0.025% MS-222 (Sigma; A5040) and euthanized via immersion in an overdose of anesthetic. For hypoxia experiments, 3 days post-fertilization (dpf) larvae were incubated at 28 °C in Danieau’s buffer in a hypoxia workstation (InvivO_2_ 300, Ruskinn) in a 5% CO_2_/95% N_2_ gas mixture to maintain 5% oxygen^36^ for 18 hours. Cardiac cryoinjuries on adult zebrafish were carried out as described previously.^37^ Briefly, fish were anesthetized and placed ventral side up in a precut sponge soaked in aquarium water containing anesthetic. A 4-mm incision was made directly above the heart to expose the ventricle, which was dried using a sterile cotton swab and a liquid nitrogen cooled probe was applied for 30 seconds. Adult fish used were aged 4 to 18 months and were randomly assigned to experimental groups. All lines were maintained according to standard procedures, and all animal work was carried out in accordance with UK Home Office and local University of Bristol regulations.

### Imaging

Larval and adult zebrafish were anesthetized and mounted in 1% low-gelling agarose (Sigma). Live imaging was performed on a ZEISS Lightsheet Z.1 system with a 40× W Plan Apochromat objective or a Leica TCS SP8 AOBS confocal laser scanning microscope with a 25×/0.95 W HC FLUOTAR objective with resonant scanner (frame intervals of 0.02–0.04 seconds). To image free particle movement in larvae, synthetic EVs containing a Cy5 conjugated microRNA (cel-miR-39-3p; not present in zebrafish^38^) were microinjected into the pericardial space. Adult hearts were dissected, fixed in 4% paraformaldehyde, and embedded and imaged as above with the conventional confocal scanner. Images were processed using Fiji,^39^ IMARIS (version 9.5.0, Oxford Instruments, United Kingdom), and Huygens Professional (version 16.10, Scientific Volume Imaging, The Netherlands). Deconvolved images are noted in the figure legends. For manual counts of EVs, all analysis was blinded and positive events were counted from 1-minute videos of the peripheral circulation.

### Cell Dissociation and EV Isolation

Adult ventricles (atria and bulbus arteriosus removed) or 4 to 6 dpf whole larvae (precise n numbers provided in figure legends) were dissociated in perfusion buffer (sterile filtered PBS plus 10 mmol/L HEPES, 30 mmol/L Taurine, and 5.5 mmol/L Glucose) plus 0.25% Trypsin, 12.5 μmol/L CaCl_2_, and 5 mg/ml Collagenase II (Worthington Biochemical Corp; LS004176) for 1 hour at 32 °C. Reactions were stopped with perfusion buffer plus 10% (vol/vol) FBS and 12.5 μmol/L CaCl_2_. Dissociated cells were centrifuged at 300 *g* (2×10 minutes), 1200 *g* (2×10 minutes), 10 000 *g* (30 minutes) and passed through either a 1.0 μm or a 0.8 μm sterile filter. Crude EVs were obtained by a standardized centrifugation step at 118 000 *g* (1 hour 54 minutes)^40^ (Optima XPN Ultracentrifuge, SW 32 Ti Rotor, Beckman Coulter) and resuspended in 300 μL sterile filtered PBS. Samples were either used immediately or snap-frozen in liquid nitrogen, stored at −80 °C and later thawed at 37 °C for 2 minutes before use.

### Dynamic Light Scatter and Nanoparticle Tracking Analysis

Hydrodynamic particle size of crude EV samples in PBS were measured in triplicate using a Zetasizer Nano-ZS (Malvern Instruments, Malvern Hills, United Kingdom). Particle concentration and size distribution were determined using a ZetaView nanoparticle tracking analysis system (Particle Metrix, Germany) and ZetaView software (version 8.05.11 SP1). Hundred nanometer polystyrene standard particles were used for calibration measurements. For video acquisition: sensitivity=85, shutter speed=100, acquisition=30 frames per second. Each sample was measured at 11 different positions, with 2 cycles of readings at each position. After automated analysis and removal of any outliers from the 11 positions, the size (diameter in nm) and the concentration (particles/mL) were calculated.

### Flow Cytometry

From crude EV samples, intact EVs were labeled with calcein violet 450 am (eBioscience; 65-0854-39) as previously described.^41^ Detergent treated samples had 0.05% Triton X-100 (Sigma; T8787) added. Flow cytometry analysis and fluorescence-activated vesicle sorting was performed on a BD Influx system, with optimizations to allow for the reliable detection of EVs.^42^ Briefly, 200 mW 488 nm (small particles and GFP), 50 mW 405 nm (calcein 450), and 100 mW 552 nm (mCherry) lasers were used with bandpass filters: 530/40, 460/50, and 610/20 nm. A small-particle detector provided high sensitivity in detecting forward scatter and a 0.45 threshold on a logarithmic scale was used. A 4-mm obscuration bar optimally detected submicron particles. For flow cytometry of EVs, a 100 μm nozzle and 21 PSI was used. To increase speed and throughput for fluorescence-activated vesicle sorting, a 70 μm nozzle and 42.9 PSI was used. EVs were sorted into 100 μL PBS. All flow cytometry experiments were performed at 4 °C.

Imaging flow cytometry analysis was carried out as previously described.^43^ Briefly, a fully calibrated (ASSIST tool) ImageStream^x^Mk II (Amnis-Luminex, SA) with 405/488 nm excitation lasers, a 785 nm side scatter laser, brightfield illumination, and a 6-channel charge-coupled device camera with time delay integration was used. For maximum resolution and sensitivity, fluidics were set at low speed, magnification at 60× (0.3 mm^2^/pixel) and 1.5 μm diameter, carboxylated polystyrene microspheres (Speed beads) were run continuously during data acquisition. Data analysis was performed using ImageStream Data Exploration and Analysis Software (IDEAS 6.2 EMD Millipore, SA).

### Electron Microscopy

Samples were prepared for cryo-EM as above with 2 adaptations; crude fractions were not filtered before the 118 000 g step to allow the full range of native EVs to be included, and the final EV pellet was resuspended in 100 μL sterile filtered PBS. Three microliters of isolated EV sample was combined with 3 μL of 10 nm fiducials and placed onto glow-discharged, 300 mesh, lacey carbon grids and blotted for 2 to 3 seconds before being plunge frozen. Grids were visualized on a FEI Tecnai 20 200 kV LaB6TEM and images acquired using a FEI Ceta 4k x4k CCD detector.

Samples were immunogold labeled and prepared for transmission electron microscopy as follows; crude EV samples were diluted 1:16 and 3 μL of this sample was placed onto pioloform grids and the buffer removed with blotting paper. The grid was fixed in 4% paraformaldehyde, washed briefly in PBS, permeabilized in 0.05% saponin, blocked in 1% BSA and 0.01% saponin, incubated with anti-RFP (MBL International Corporation; PM005, 1:20), washed in PBS, incubated with anti-Rabbit 6 nm gold particles (Aurion; 806.011, 1:20), and washed in PBS. Grids were negatively stained in 0.3% uranyl acetate and 1.8% methyl cellulose (Sigma; M6385) on ice, air-dried and visualized on a FEI Tecnai 12 120 kV BioTwin Spirit Transmission electron microscopy and images acquired using a FEI Eagle 4k x4k CCD camera.

### Sucrose Density Gradient

Crude EV samples isolated from whole larvae were floated into a sucrose (VWR; 443815S) density gradient. Sucrose gradients were prepared as previously described.^41^ Briefly, they were layered in 38.5 mL, Open-Top Thinwall Ultra-Clear Tubes (Beckman Coulter; 344058) and EVs were floated by centrifugation at 179 500 *g* (20 hours) (Optima XPN Ultracentrifuge, SW 32 Ti Rotor, Beckman Coulter). EVs were obtained from combined sucrose concentrations as follows: 1.543 to 1.657 M (F1), 1.314 to 1.429 M (F2), 1.086 to 1.200 M (F3), 0.857 to 0.971 M (F4), 0.629 to 0.743 M (F5) and 0.400 to 0.514 M (F6). Fractions 1-6 (F1-6) were individually diluted 1:10 in sterile filtered PBS and EVs were obtained by centrifugation at 118 000 *g* (1 hour 54 minutes) (Optima XPN Ultracentrifuge, SW 32 Ti Rotor, Beckman Coulter).

### Protein Analysis

Western blot and dot blot analyses were carried out using standard methods. Briefly, cells, and EV pellets were lysed on ice with lysis buffer (125 mmol/L NaCl, 20 mmol/L TRIS pH 7.4, 1% Nonidet P40 (Sigma-Aldrich; 11754599001), 10% Glycerol, 0.1 mg/mL Phenylmethylsulfonyl fluoride, protease cocktail inhibitor (Sigma-Aldrich; 04693159001), 50 mmol/L NaF and 10 mmol/L Na_3_O_4_V. For Western blots, equal protein concentrations were resolved via 8% SDS–PAGE and transferred to Immobilon-P polyvinylidene difluoride membrane (Sigma-Aldrich; IPVH00010). For dot blots, protein was spotted directly onto Immobilon-P polyvinylidene difluoride membrane (Sigma-Aldrich; IPVH00010). Antibodies used: anti-ALIX (Sigma-Aldrich; SAB4200476, 1:500), anti-CD63 (Santa Cruz Biotechnology; sc-15363, 1:200), anti-GAPDH (proteintech; 60004-1-IG, 1:5000), anti-GFP (proteintech; 66002-1-IG, 1:2000), anti-RFP (MBL International Corporation; PM005, 1:1000), and anti-SYNTENIN (ThermoFisher Scientific; PA5-42592, 1:800), Goat anti-Rabbit IgG (H+L) Cross-Adsorbed Secondary Antibody, HRP (ThermoFisher Scientific; A16104, 1:10000) and Donkey anti-Mouse IgG (H+L), Cross-Adsorbed Secondary Antibody, HRP (biotium; 20404, 1:10000). ECL Western Blotting Substrate (ThermoFisher Scientific; 32109) or SuperSignal West Femto Maximum Sensitivity Substrate (ThermoFisher Scientific; 34094) was added before being detected using a Syngene G:BOX Chemi-XT4.

### Statistics

In all cases n numbers refer to biological replicates unless otherwise stated. All experiments were repeated at least twice. GraphPad Prism6/7 was used for raw data recording/analysis. Statistical tests used were nonparametric Mann-Whitney or Kruskal-Wallis/Dunn’s multiple comparison tests or a randomized permutation test using total variation distance between 2 groups (details in figure legends). All statistical analysis was blinded. In all cases, error bars represent SD. For all data sets, a Grubb’s outlier test was performed and any significant outliers (alpha=0.05) were removed. *P* are included in all graphs.

## Results

### Stable Labeling and Imaging of Endogenous EVs In Vivo

Despite recent advances in labeling methods, the distribution and function of cell type specific EVs in vivo remains incompletely understood. To label large proportions of EVs produced by individual cell types and avoid targeting sub-populations via EV enriched protein labeling, we hypothesized that stable transgenic zebrafish lines expressing cell membrane-tethered fluorophores would also label EVs produced by those cells (Figure IA in the Data Supplement). Similar unbiased inner leaflet cell-membrane labeling techniques have been used to label cells and their derived EVs in other models.^25,44^ To demonstrate that endogenous EVs could be labeled with this system, we initially used a transgenic line where prenylated GFP is driven by a near-ubiquitous promoter, tethering the fluorophore to the inner leaflet of the plasma membrane (Tg(*Ola.Actb:Hsa.HRAS-EGFP*) (referred to as Tg(*actb2:HRAS-EGFP*)); Figure IA and IB in the Data Supplement^29^). Live imaging of Tg(*actb2:HRAS-EGFP*) larvae revealed subcellular GFP+ particles in the peripheral circulation and in the pericardial space at 3 dpf (Figure IC and IG and Movies I and II in the Data Supplement). The pericardial space is a relatively large extracellular space in larval zebrafish (Figure ID through IF in the Data Supplement) and GFP+ EVs were observed moving within the pericardial fluid, influenced by the movement of the heart (Movie II in the Data Supplement). Additionally, we used a ubiquitously expressed secreted Annexin-V line, which binds with high affinity to the phosphatidylserine expressed on the outer surface of apoptotic cells (Tg(*tbp:GAL4*);(*UAS:secA5-YFP*))^30^; Figure IIA and IIB in the Data Supplement), but also on populations of EVs, including those relevant in cardiovascular disease.^45,46^ As with the *actb2* line, we observed Annexin-V labeled particles in both the peripheral circulation and pericardial space of larval zebrafish (Figure IIC and IID and Movie III in the Data Supplement).

**Figure 1.**
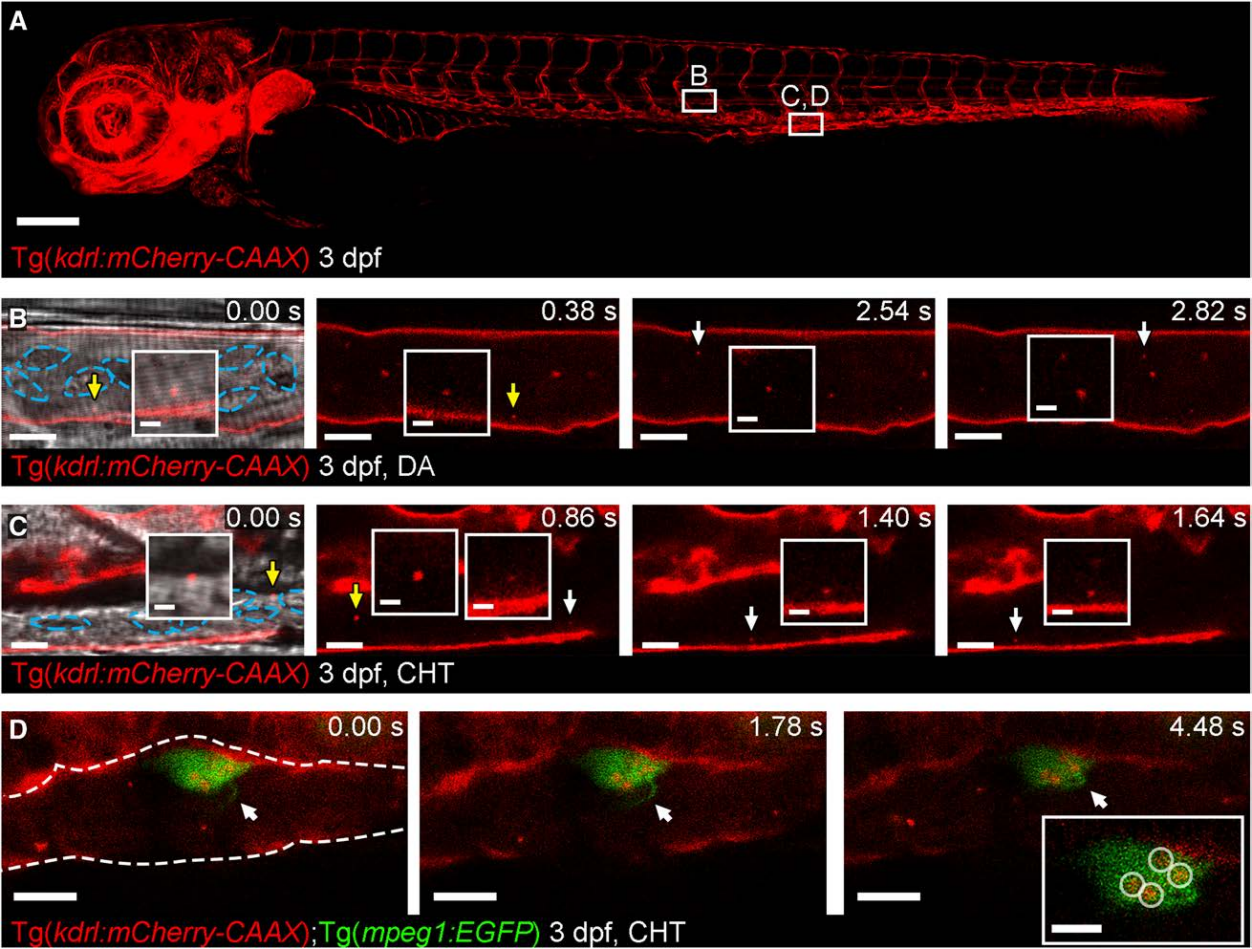
**Cell-type specific extracellular vesicle (EV) labeling strategy and live imaging in the peripheral circulation.A**, Overview image of a Tg(*kdrl:mCherry-CAAX*) fish at 3 dpf. All endothelial cells are labeled with mCherry. The boxed areas define the regions shown in the image sequence in **B**, **C**, **D**. **B**, Image sequence of mCherry+ endothelial cell (EC)-EVs moving through the dorsal aorta (DA; arrows and inset). The resolution of light-based detection methods limits our ability to accurately determine the size of endogenous EVs during live imaging. **C**, Image sequence of mCherry+ EC-EVs moving through the caudal hematopoietic tissue (CHT; arrows and inset). **D**, Image sequence of a macrophage (green) in the CHT of a Tg(*kdrl:mCherry-CAAX*);Tg(*mpeg:EGFP*) fish at 3 dpf, a macrophage protrusion moves toward the cell body (arrows) and intracellular compartments contain mCherry+ EC-EVs (circled in inset). The blue dashed lines in **B**, **C** demark blood cells. The white dashed line in **D** demarks the endothelium. Anterior is to the left. Scale bars: **A**, 200 μm; **B** and **C**, 5 μm; insets in **C**, 2 μm; **D**, 10 μm; insets in **D**, 5 μm.

**Figure 2.**
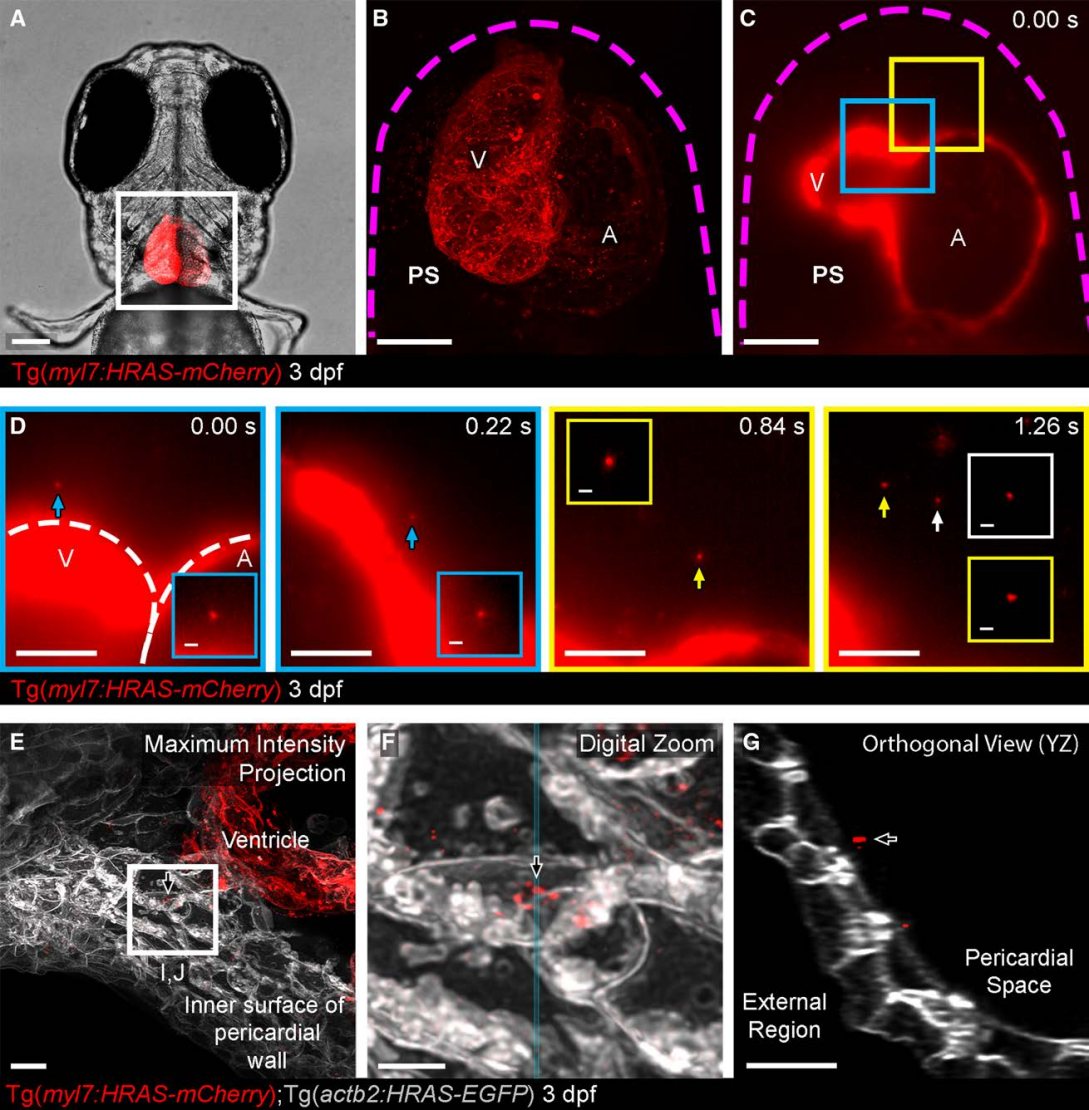
**Cell-type specific extracellular vesicle (EV) labeling strategy and live imaging in the pericardial space (PS).A**, Ventral view of 3 dpf Tg(*myl7:HRAS-mCherry*) zebrafish, boxed region highlights position of **B** and **C**. **B** and **C**, Overview images of the entire hearts of Tg(*myl7:HRAS-mCherry*) fish in ventral view. **B**, A maximum projection of a fixed fish; (**C**) a single plane of live light sheet imaging. **D**, Image sequences of higher magnification views of the color coded boxed areas in **C**. mCherry+ CM-EVs are observed moving through the pericardial space (arrowed and inset). **E–G**, A maximum intensity projection of deconvolved images of the ventricle and internal surface of the pericardial wall (**E**) reveals static CM-EVs as shown with digital zoom of boxed region (**F**). The orthogonal view (YZ) of this region suggests the EVs may be associated with a layer of unmarked ECM (extracellular matrix) rather than direct contact with underlying cells (**G**). The magenta dashed line in **B** and **C** demarks the pericardial wall. The white dashed line in **D** demarks the ventricle (V) and atrium (A). Arrows indicate static EVs. Anterior is to the left. Scale bars: **A**, 100 μm; **B** and **C**, 50 μm; **D**, 20 μm; insets in **D**, 2 μm; **E** and **G**, 5 μm; **F**, 2 μm.

Although these transgenic lines suggested we could successfully identify endogenous EVs in vivo, they do not allow us to determine the cellular origin of these subcellular particles. We therefore made use of 2 more transgenic lines that express a prenylated fluorophore driven by cell specific, cardiovascular relevant promoters, whereby the fluorophore is incorporated into the EVs in a cell-type specific manner (Figures 1 and 2). Live confocal or light-sheet imaging of larval zebrafish expressing an endothelial specific promoter driving membrane-tethered mCherry (Tg(*kdrl:mCherry-CAAX*)^31^); revealed the presence of fluorophore-labeled subcellular particles (referred to as EC-EVs) in the peripheral circulation (Figure IA through ID and Movie IV in the Data Supplement). Putative EC-EVs moved rapidly in the peripheral circulation with the blood flow and were observed both in arterial flow in the dorsal aorta (mean=63±23 [SD] EVs/minute) and in venous flow in the caudal haematopoietic tissue (mean=333±33 [SD] EVs/minute) (Figure 1B through 1D and Movie IV in the Data Supplement). Dual imaging with brightfield confirmed that these EC-EVs moved independently from cells in the blood (Figure 1B and 1C and Movie IV in the Data Supplement). It has been suggested that macrophages of the innate immune system can receive EVs from multiple cell types^24,28^ and as macrophages are present in the peripheral circulation of larval zebrafish we investigated if they may receive EC-EVs in our model. Live imaging of Tg(*kdrl:mCherry-CAAX*); Tg(*mpeg1:EGFP*) double transgenic fish (GFP labeling macrophage cytoplasm) reveals transfer of EC-EVs to intravascular macrophages, which were observed making protrusions into the lumen of vessels, potentially to capture passing EVs (Figure 1D and Movie V in the Data Supplement), as previously shown for other EV populations.^24,28^

Live imaging of the heart of 3 dpf larvae with a cardiomyocyte-specific promoter driving membrane tethered mCherry (Tg(*myl7:HRAS-mCherry*)^35^) revealed mCherry+ particles (referred to as cardiomyocyte-derived EV [CM-EVs]) moving within the pericardial fluid, as observed for Tg(*actb2:HRAS-EGFP*) labeled EVs (Figure 2D and Movie VI in the Data Supplement). This movement is similar to that described for free proepicardial cells, which dissociate from the dorsal pericardial wall and move, influenced by the heartbeat, within the pericardial fluid during epicardial formation.^47^ This process of free proepicardial cell movement is largely complete by 3 dpf,^47^ and our own data suggest the pericardial space is devoid of free moving cells at this stage ruling out interactions of these EVs with cells (Figure IIIA through IIID and Movie VII in the Data Supplement). Additionally, we injected fluorescent ≈50 nm synthetic nanoparticles^38^ into the pericardial space of 3 dpf larvae and observed similar stochastic movement confirming the EVs were free moving (Figure IIIE and Movie VIII in the Data Supplement). Some static CM-EVs were observed in proximity to the pericardial wall but not within cells, suggesting they may be passively or actively associated with regions of extracellular matrix (Figure 2E through 2G).

### Validation of Endogenous EVs

**Figure 3.**
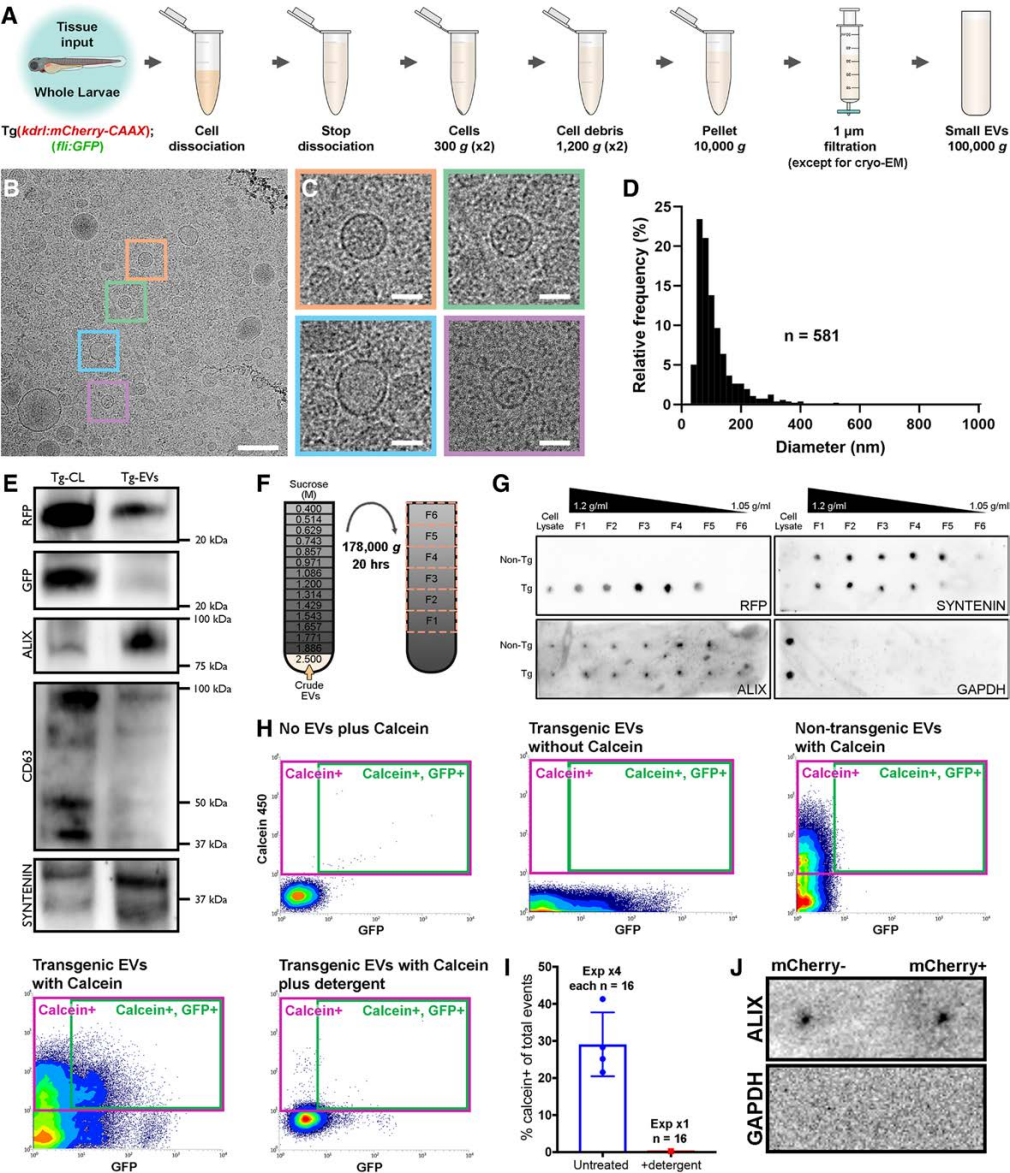
**Validation of endogenous cardiovascular extracellular vesicles (EVs) from larval zebrafish.A**, Schematic describing the centrifugation steps taken to isolate EV fractions following cell dissociation of whole zebrafish larvae. **B** and **C**, Cryo-EM micrograph of isolated EVs from a pool of whole 3 dpf larvae (n=1200). **C**, A panel of 4 higher magnification views of the boxed regions in **B**. **D**, Histogram of the size distribution of EVs visualized by cryo-EM. n refers to number of EVs analyzed. **E**, Western blot analysis of protein extracted from the cells (Tg-CL) and crude EV fraction (Tg-EVs) isolated from Tg(*kdrl:mCherry-CAAX*); Tg(*fli:GFP*) 6 dpf larvae (n=600). **F**, Schematic describing the sucrose gradient approach used to more precisely isolate EVs from the crude EV fraction. **G**, Dot blot analysis of protein extracted from the cells and sucrose gradient fractions isolated from nontransgenic (Non-Tg [n=600]) and Tg(*kdrl:mCherry-CAAX*) (Tg [n=600]) 6 dpf larvae. **H**, Typical flow cytometry scatter plots showing the gates used to sort EVs and the controls used to define these gates: An extraction buffer only control plus calcein AM reveals background noise, EVs extracted from Tg(*actb2:HRAS-EGFP*) fish without calcein AM indicates GFP+ EVs, EVs extracted from nontransgenic (wildtype) fish and labeled with calcein AM defines a calcein+ gate and analysis of EVs extracted from Tg(*actb2:HRAS-EGFP*) fish with calcein AM labeling identifies a gate of GFP+ calcein+ EVs. Treatment of transgenic EVs labeled with calcein AM plus detergent destroys the majority of EVs, confirming their lipidaceous structure. Similar gating strategies can be used to analyze mCherry+ EVs from Tg(*kdrl:mCherry-CAAX*) and Tg(*myl7:HRAS-mCherry*) fish. **I**, Plot of the number of calcein+ EVs of total events from untreated and detergent treated samples. **J**, Dot blot analysis of protein extracted from FAVS isolated particles, sorted for both mCherry- and mCherry+ EVs from Tg(*kdrl:mCherry-CAAX*) 6 dpf larvae, confirms expression of the EV component Alix and absence of Gapdh expression. Scale bars: **B**, 200 nm; **C**, 50 nm.

To allow additional ex vivo validation of endogenous vesicles from entire zebrafish larvae we employed a tissue dissociation, differential centrifugation and filtration protocol to generate a crude overall EV population (Figure 3A). To determine the precise diameter, morphology and overall size distribution of total endogenous zebrafish EVs, we used cryo-EM to analyze extracted vesicles (Figure 3B through 3D). Cryo-EM revealed vesicular particles which ranged in size from 23 nm to 808 nm with a mean diameter of 103 nm (Figure 3B through 3D). Cryo-EM analysis also revealed EVs with multiple different morphologies that are remarkably similar to those described for other species and cell types (Figure IVA and IVB in the Data Supplement^48,49^). Next, we analyzed total EVs for protein expression of RFP and known EV components (Figure 3E through 3G). Cells from Tg(*fli:EGFP*) (cytoplasmic GFP labeling ECs); Tg(*kdrl:mCherry-CAAX*) larvae expressed high levels of RFP and GFP whereas EVs were highly positive for the membrane tethered RFP but retained limited GFP (Figure 3E). EVs expressed high levels of known exosomal markers Alix, Syntenin and, to a lesser degree, CD63 (Figure 3E). We next used a sucrose gradient to separate the crude total EVs into 6 separate density fractions (Figure 3F). RFP was absent from cells and EVs extracted from nontransgenic fish but was present in EVs from Tg(*kdrl:mCherry-CAAX*) larvae and was enriched in fractions F3 and F4 where the majority of EVs are found (1.11–1.16 g/mL; Figure 3G^42,50^). EV markers Alix and Syntenin were found in all fractions except the least dense whereas Gapdh was only detected in the cell lysate (Figure 3G).

To allow us to obtain and verify our endogenous fluorescently labeled EVs, we used a modified flow cytometer to analyze ubiquitous (*actb2*(GFP)+) EVs extracted from whole zebrafish larvae at 3 dpf (Figure 3H), using similar methods to those previously described.^42^ Isolated EVs were labeled with calcein violet 450 am, which when converted to a fluorescent form by intravesicular esterases becomes membrane impermeable and so labels only intact vesicles,^41^ before analysis by flow cytometry. Total populations of calcein+ and *actb2*(GFP)+ EVs were assessed from EVs extracted from pools of nontransgenic and Tg(*actb2:HRAS-EGFP*) larvae (Figure 3H). Control experiments of singly labeled EVs (GFP or calcein) from transgenic and nontransgenic zebrafish allowed stringent double positive (ie, cell type specific [fluorophore+] and intact [calcein+]) EV specific gates to be assigned, avoiding any background signal (Figure 3H). Analysis of EV fractions following detergent treatment confirmed their lipidaceous nature (Figure 3H and 3I). Flow cytometry analyses suggest that 29%±8.6% (SD) of total events are labeled with calcein, representing intact EVs containing esterases (Figure 3H). Similarly, flow cytometry analysis suggests that 30%±6.9% (SD) of calcein+ vesicles are also *actb2*(GFP)+ (Figure 3H). Although sizing of EVs via flow cytometry is inaccurate without precise instrument calibration,^51^ analysis of the forward scatter profile reveals similar size distributions to those observed by cryo-EM (Figure VA in the Data Supplement). We also analyzed calcein+ EVs extracted from Tg(*actb2:HRAS-EGFP*) larvae using an imaging flow cytometry ImageStream^x^Mk II system, confirming the presence of individual single or double labeled vesicles (Figure VC and VD in the Data Supplement). Imaging flow cytometry suggests 65% of total events are calcein(violet)+ (concentration as per routine 300 μL resuspension volume=2.14×10^8^ EVs/mL) demonstrating the enhanced sensitivity and value of the ImageStream system.^52^ This analysis suggests 34% of calcein+ vesicles are also *actb2*(GFP)+, supporting our previous flow cytometry assessments. Protein expression analysis confirmed the presence of Alix in sorted mCherry+ EC-EVs and mCherry- EVs from Tg(*kdrl:mCherry-CAAX*) larvae whereas Gapdh served as a negative control (Figure 3J).

Collectively, our data describe methods to label cell-type specific EVs in vivo and highlights larval zebrafish as an ideal model to investigate EV function in multiple different cardiovascular functions and pathologies.

### Tissue Injury Models Affect Endogenous EVs In Vivo

We next sought to demonstrate the utility of this labeling system to observe changes to endogenous vesicle production during pathological states. As tissue hypoxia is an integral component of ischemic injury and has been shown to induce release of EC-EVs primarily in vitro,^53^ we used our model to investigate this in vivo. Incubating 3 dpf Tg(*kdrl:mCherry-CAAX*) larvae in 5% oxygen for 18 hours significantly increased the number of EC-EVs observed in the peripheral circulation when compared with control larvae maintained in normoxic conditions (Figure VIA through VIC and Movie IX in the Data Supplement). This suggests that a global ischemic/hypoxic environment increases EC-EV release into the peripheral circulation indicating dynamic EV responses following noninvasive tissue challenge in larval zebrafish. This further demonstrates the potential of the larval zebrafish model to assess EV function in pathological states in vivo. To further address the full complexity of endogenous cardiovascular EVs released following localized cardiac injury in fully differentiated tissues, we next investigated EVs in adult zebrafish before and after a well-established model of cardiac injury.^37^

### Characterization of EVs From Adult Cardiac Tissue

**Figure 4.**
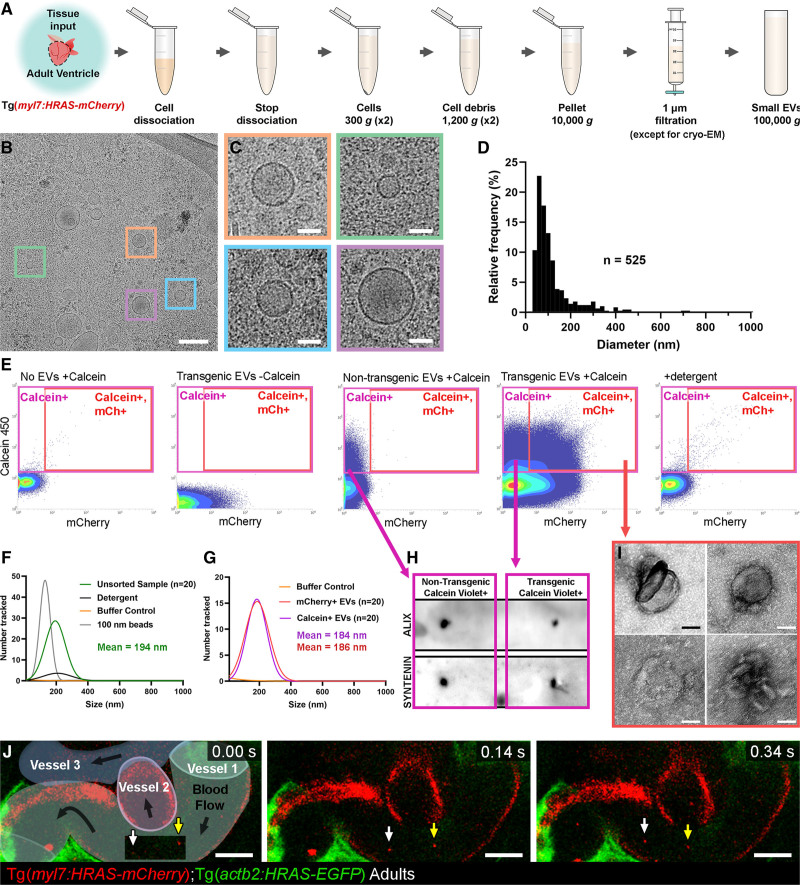
**Validation of endogenous cardiovascular extracellular vesicles (EVs) from adult zebrafish.A**, Schematic describing the centrifugation steps taken to isolate EV fractions following cell dissociation of adult zebrafish ventricular tissue. **B** and **C**, Cryo-EM micrograph of isolated EVs from a pool of ventricles (n=60). **C**, A panel of 4 higher magnification views of the boxed regions in **B**. **D**, Histogram of the size distribution of EVs visualized by cryo-EM. n refers to number of EVs analyzed. **E**, Typical flow cytometry scatter plots showing the gates used to sort adult cardiac EVs and the controls used to define these gates. **F** and **G**, Gaussian distribution of NTA analysis on unsorted EV fractions before and after detergent treatment (**F**) and after sorting for calcein+ EVs and mCherry+ calcein+ EVs (**G**). **H**, Dot blot analysis of protein extracted from FAVS isolated particles, sorted for calcein+ EVs from both nontransgenic and Tg(*myl7:HRAS-mCherry*) adult ventricular tissue, confirms expression of the EV components Alix and Syntenin. **I**, TEM-negative stain micrographs of FAVS isolated particles, sorted for mCherry+ calcein+ EVs from Tg(*myl7:HRAS-mCherry*) adult ventricular tissue. **J**, Schematic overlay describing the position of the 3 vessels visible in the integrated time series of live imaging of endothelial cell-EVs in the peripheral circulation of an adult Tg(*actb2:HRAS-EGFP*); Tg(*kdrl:mCherry-CAAX*) double transgenic fish. White and yellow arrows indicate 2 EC-EVs moving with the blood flow. Scale bars: **B**, 200 nm; **C** and **I**, 50 nm; **J**, 10 μm.

To characterize endogenous EVs from adult cardiac tissue for the first time, we initially extracted total populations and analyzed them via cryo-EM (Figure 4A through 4D). As for larvae, we observed EVs with different sizes and morphologies (21–841 nm, 119 nm mean diameter; Figure 4B through 4D and Figure IV in the Data Supplement). Interestingly, although similar to larval EVs, adults exhibited a larger proportion of small, single EVs (Figure IVC in the Data Supplement). Next, we extracted calcein+ and CM-EVs from isolated ventricles of uninjured adult Tg(*myl7:HRAS-mCherry*) fish and analyzed these ex vivo using similar techniques described for larval samples (Figure 4E). Flow cytometry analysis revealed populations of intact, cell-type specific CM-EVs derived from adult ventricles that could be obtained in similar proportions but much higher numbers than EVs from entire larval fish (Figure 4E; Figure VB in the Data Supplement). Nanoparticle tracking analysis demonstrated calcein+ and CM-EVs with similar size ranges before and after fluorescence activated vesicle sorting with this analysis further confirming their lipid composition (Figure 4F and 4G). Sorted calcein+ EVs expressed Alix and Syntenin (Figure 4H) and transmission electron microscopy revealed classic EV morphologies of sorted calcein+ mCherry+ CM-EVs from the same experiment (Figure 4I). Additionally, immunogold labeling of RFP of crude EVs extracted from Tg(*myl7:HRAS-mCherry*) ventricles reveals unlabeled and labeled vesicles of expected sizes (Figure VII in the Data Supplement). Finally, to determine if cell type specific EVs could be visualized in vivo in adults, we performed live imaging of superficial vessels of adult Tg(*kdrl:mCherry-CAAX*) fish revealing EC-EVs present in the peripheral circulation (Figure 4J and Movie X in the Data Supplement).

### Exchange of EVs Differs Between Cardiac Cell Types

**Figure 5.**
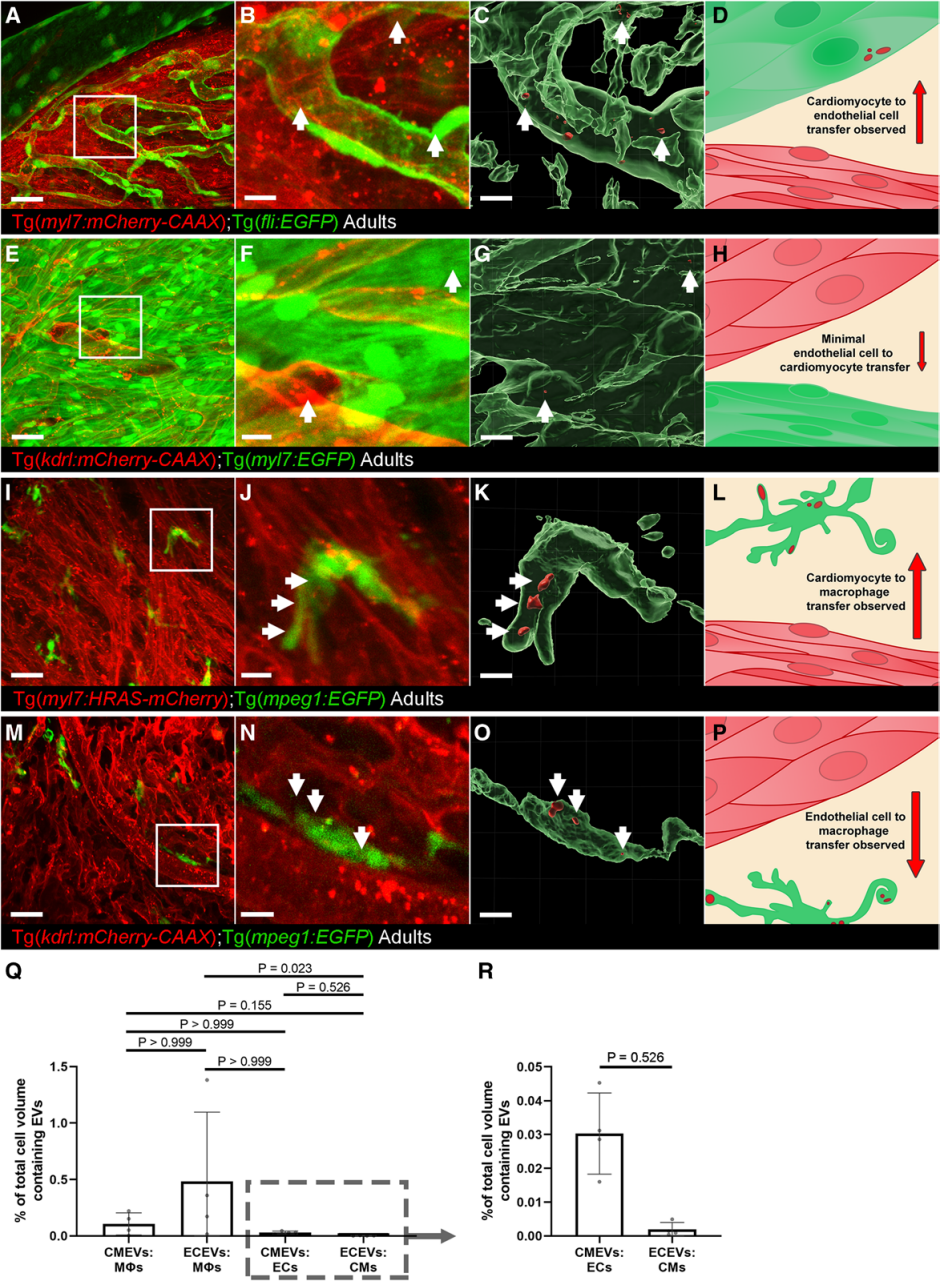
**Whole-mount high-resolution imaging of fixed adult hearts to characterize extracellular vesicle (EV) transfer to recipient cells.A–D**, Cardiomyocyte (CM)-EV transfer to endothelial cells (ECs). **A**, Overview maximum projection showing the surface view of an adult Tg(*myl7:HRAS-mCherry*); Tg(*fli:EGFP*) zebrafish ventricle, boxed region highlights position of **B** and **C**. **B** and **C**, Digital zoom of the boxed region in **A**. **B**, A maximum projection; **C**, Three-dimensional reconstruction of the same image reveal CM-EVs within ECs (CMs removed for clarity). **D**, Schematic summary depicts localization of CM-EVs within ECs. **E–H**, Limited EC-EV transfer to CMs. **E**, Overview maximum projection showing the surface view of an adult Tg(*kdrl:mCherry-CAAX*); Tg(*myl7:EGFP*) zebrafish ventricle, boxed region highlights position of **F** and **G**. **F** and **G**, Digital zoom of the boxed region in **E**. **F**, A maximum projection; **G**, A 3-dimensional reconstruction of the same image reveal EC-EVs within CMs (ECs removed for clarity). **H**, Schematic summary depicts localization of EC-EVs within CMs. **I–L**, CM-EV transfer to macrophages. **I**, Overview maximum projection showing the surface view of an adult Tg(*myl7:HRAS-mCherry*); Tg(*mpeg:EGFP*) zebrafish ventricle, boxed region highlights position of **J** and **K**. **J** and **K**, Digital zoom of the boxed region in **I**. **J**, A maximum projection; **K**, A 3-dimensional reconstruction of the same image reveal CM-EVs within macrophages (CMs removed for clarity). **L**, Schematic summary depicts localization of CM-EVs within macrophages. **M–P**, EC-EV transfer to macrophages. **M**, Overview maximum projection showing the surface view of an adult Tg(*kdrl:mCherry-CAAX*); Tg(*mpeg:EGFP*) zebrafish ventricle, boxed region highlights position of **N** and **O**. **N** and **O**, Digital zoom of the boxed region in **M**. **N**, A maximum projection; **O**, A 3-D reconstruction of the same image reveal EC-EVs within macrophages (ECs removed for clarity). **P**, Schematic summary depicts localization of EC-EVs within macrophages. **Q** and **R**, Volume measurements of EV containing compartments are shown as a percentage of volume measurements of the cells within the field of view from 3D image analysis. The boxed region in Q highlights the expanded view in R. Arrows indicate EVs within recipient cells. Scale bars: **A**, **E**, **I**, **M**=20 μm; **B**, **C**, **F**, **G**, **J**, **K**, **N**, **O**=5 μm.

Next, we sought to investigate the transfer of endogenous EVs between different cell types within the cardiac microenvironment. We performed confocal imaging of hearts from combinations of transgenics allowing us to observe EV transfer between CMs, ECs and macrophages (Figure 5). Combining membrane tethered mCherry lines (cells and EVs labeled) with cytoplasmic GFP lines (only cells labeled) revealed transfer of CM-EVs to ECs (Figure 5A through 5D) but indicated only very limited transfer of EC-EVs to CMs (Figure 5E through 5H). Conversely, transfer of both CM-EVs and EC-EVs to macrophages resident in the heart was observed (Figure 5I through 5P and Movie XI in the Data Supplement). Quantification of the degree of transfer between different cell types suggests the majority of this labeled material is received by macrophages, followed by ECs (Figure 5Q and 5R), with the lowest transfer observed to CMs (Figure 5R). These data reveal new insights into cellular communication occurring during homeostasis in vivo between different cardiovascular cell types and demonstrate the utility of the labeling method to investigate complex cellular processes in different tissues.

### Cardiovascular EVs Exhibit Dynamic Responses to Cardiac Injury

**Figure 6.**
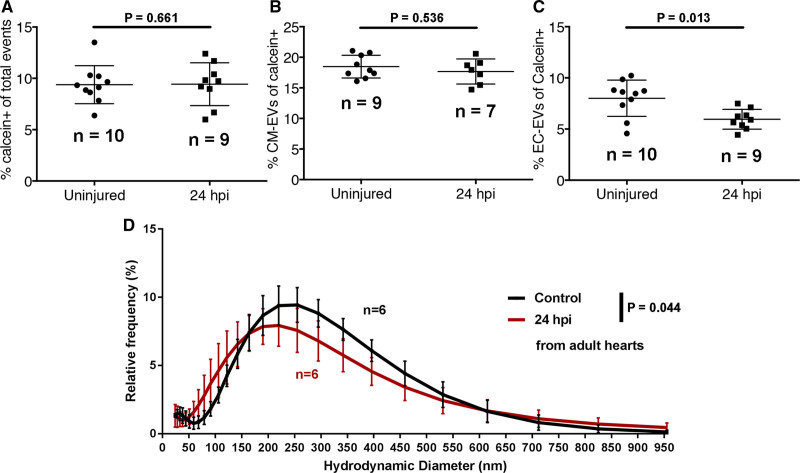
**A model of myocardial infarction (MI) induces dynamic changes in cardiovascular extracellular vesicles (EVs) from adult zebrafish. A–C**, Quantification of the number of calcein+ (**A**), *myl7*(mCherry)+ (**B**), and *kdrl*(mCherry)+ EVs in uninjured and injured hearts at 24 hpi. **D**, Histogram of DLS analysis reveals a significant shift in the size of overall EVs at 24 hpi compared with uninjured hearts. Statistical analysis in **A–C**, 2-tailed Mann-Whitney *U* tests. Statistical analysis in **D**: a custom permutation test using total variation distance was used to test the null hypothesis that control and 24 hpi distributions were the same.

As EVs are linked to the progression of cardiovascular disease after myocardial injury,^54^ we next assessed the EV response to tissue damage and further demonstrate the value of the adult model. We performed cardiac cryoinjury on adult zebrafish and extracted EVs from isolated ventricles of unwounded and 24 hours post-injury (hpi) fish and analyzed them via flow cytometry and dynamic light scatter (Figure 6). Flow cytometry did not reveal significant changes to the overall number of calcein+ or intact CM-EVs in the heart following cardiac injury (Figure 6A and 6B). By contrast, there were significantly fewer EC-EVs as a proportion of calcein+ EVs (Figure 6C), suggesting an increase in EVs derived from additional cell types, for example, interstitial fibroblasts or inflammatory cells, which were not assessed here. Interestingly, dynamic light scatter revealed a significant shift in the size distribution of total EVs at 24 hours post-injury when compared with unwounded hearts (Figure 6D) suggesting a dynamic EV response following cardiac injury.

## Discussion

Cell-cell communication roles for EVs have been mostly studied in ex vivo and in vitro cell models. However, relatively little is known about the in vivo function of native EVs. Here, we have described methods to fluorescently label cell-type specific endogenous EVs and visualized these in vivo in a vertebrate zebrafish model system. Our findings complement recent studies that describe the labeling, biodistribution, and potential functional roles of exogenous tumor derived and endogenous CD63+ EVs in larval zebrafish.^24,28^ Additionally, we have provided the first cryo-EM assessment of total larval and adult zebrafish cardiac EVs, which reveals size distributions reminiscent of EVs from other species and cell types^48,49^ and highlights the plethora of different EV morphologies found in vivo. The described techniques and advancements in the use of zebrafish for in vivo EV research pave the way for investigations into homeostatic and pathophysiological states^55^ and the involvement of endogenous EVs in cellular processes such as tissue regeneration.

The labeling strategy described here allows the identification of the cellular origin of EVs and their visualization in vivo without reliance on the expression of specific marker proteins. As the resolution and point spread function of light-based detection methods limit our ability to accurately size the endogenous EVs during live imaging, we have analyzed these particles ex vivo to determine their true size. EM and nanoparticle tracking analysis analyses of sorted vesicles reveals expected sizes, and protein expression of EV markers suggests labeling of exosomes and potentially microvesicles. Additionally, our cryo-EM analyses of total EVs suggests limited vesicles >300 nm in diameter suggesting those we observe via live imaging are indeed smaller vesicles that seem larger because of imaging limitations, although we cannot rule out the possibility of aggregated EVs traveling together. Currently, the proportion of EVs that incorporate fluorophore labeling of sufficient brightness during biogenesis is incompletely understood; however, our data support an increasing body of evidence that suggests passive fluorophore membrane tethering strategies can successfully identify different EV subtypes with efficiencies similar to protein fusion labeling.^25,44,56–58^

We have chosen to use a stringent gating strategy for flow cytometry assessments of fluorophore labeled EVs, relying on the retention of fluorescent calcein, which has been shown to selectively reveal EVs with intact membranes when activated by intravesicular esterases.^41^ Our gating strategies suggest that ≈30% to 65% of total singlet events or fluorescent (GFP+ or mCherry+) vesicles are labeled with calcein, indicating relatively restricted esterase incorporation in zebrafish EVs and/or limited survival of intact EVs following isolation protocols. Therefore, the use of calcein labeling limits the number of EVs that can be taken forward for further analysis but remains an important step in guaranteeing the integrity of these vesicles. Nevertheless, regardless of the described limitations, shared fluorescence between the selected cell types and EVs observed within extracellular space confirms the cellular origin of these vesicles and sufficient numbers of intact vesicles can be obtained for further studies. Combining this system with other labeling methods will allow cell type specific sub classes of EVs to be defined in vivo in the future.

We have also shown for the first time that endogenous cardiac EVs from adult zebrafish can be visualized and extracted. Using combinations of stable transgenic lines, we can observe the conversations between different cell types in the cardiac microenvironment. Tissue-resident macrophages received the majority of labeled material from other cell types although ECs also received CM-EVs. Similarly, in larvae, EC-EVs were observed within intravascular macrophages, which were often observed making protrusions, potentially to catch passing EVs. This suggests macrophages may receive the majority of EVs during homeostasis in vivo, supporting previous findings.^24,28^ Interestingly, large numbers of EC-EVs were transferred to macrophages compared with CM-EVs (Figure 5Q) whereas flow cytometry suggested a larger proportion of CM-EVs than EC-EVs within cardiac tissue as a whole (Figure 6B and 6C), potentially suggesting targeted crosstalk between ECs and cardiac macrophages. As macrophages are known scavengers of cellular debris and dying cells, we cannot rule out transfer of material by these mechanisms, although very little cell death is observed in homeostatic hearts.^59,60^ However, transfer between CMs and ECs, nonscavenger specialist cells, suggests active transport of membrane-bound EVs. EC-EVs could be observed moving rapidly with the blood flow in superficial vessels of adult fish and transmission electron microscopy reveals a characteristic cup-shaped appearance of sorted CM-EVs from isolated adult hearts. Cryo-EM measurements and nanoparticle tracking analysis reveal similar size distributions for total EVs extracted from adult hearts and from whole larvae and are in line with previous reports of EVs from zebrafish, mice, and humans.^4,24,61–63^

Importantly, we have demonstrated that models of cardiovascular injury can induce changes to EV number and size, suggesting a dynamic intercellular communication response that involves EVs, supporting previous in vitro models, clinical findings, and studies in nonregenerative rodent models of myocardial infarction.^4,5,10,61,64–66^ Similar numbers of overall EVs and CM-EVs following cardiac injury but a reduction in EC-EVs, as a proportion of total (calcein+) EVs, suggest an increased proportion of EVs deriving from an, as yet undetermined, cell-type. Interestingly, we observed an increase in EC-EVs following larval injury models and a reduction in EC-EVs following an adult myocardial infarction model, suggesting dynamic changes to EVs that may be highly tissue- and time-point dependent. Further studies will be required to determine if the reduction in EC-EVs results from reduced numbers of ECs in the injured heart at 24 hours post-injury or from dynamic changes to EC-EV production following injury. Our data further suggest that there is a significant shift to smaller EVs at 24 hpi potentially indicating increased exosome release. This is similar to what has been described for clinical plasma samples in patients undergoing cardiac surgery^4^ and may represent an active shift in EV biogenesis in response to myocardial infarction. Additionally, further studies to define the cargo and composition of these postischemic EVs could reveal their roles in tissue repair and regeneration and identify potential therapeutic targets that could be validated in mammalian models.

In summary, we have shown that zebrafish are an ideal model to investigate endogenously produced cell-type specific EVs stably labeled with fluorophores, revealing their cellular origin and allowing them to be observed and tracked in vivo. Although challenges remain related to labeling efficiencies/brightness and current imaging capabilities impacting on detection of these small vesicles in vivo, we have begun to address the complexity of endogenous EVs in a valuable vertebrate model. Large numbers of cell-type specific EVs can be obtained from adult hearts allowing future evaluations of cargo during homeostasis and in models of disease. Finally, multiple cell types exchange EVs in the adult heart and models of ischemic injury produce dynamic changes to EV size and numbers produced by different cell types demonstrating the role of these small vesicles in these processes.

## Acknowledgments

We thank the Wolfson Bioimaging Facility for imaging expertise, Professor Dan Peer (Tel Aviv University) for providing synthetic nanoparticles, Professor Jonathan Rougier (University of Bristol) for statistical advice, Judith Mantell for EM expertise, and Helen Rice for flow cytometry support.

## Sources of Funding

This work was supported by a BHF Intermediate Fellowship (FS/15/2/31225) to R.J. Richardson, a BHF studentship for AS awarded jointly to R.J. Richardson and C. Emanueli (FS/18/34/33666), the BHF Oxbridge Centre of Regenerative Medicine (RM/13/03/30159) (jointly to R.J. Richardson and C. Emanueli), a BHF Chair award grant (CH/15/31199) and Leducq Transatlantic Network MIRVAD (both to C. Emanueli), Wellcome Trust funding of a ZEISS Lightsheet Z.1 system and MRC funding of a preclinical in vivo functional imaging platform for translational regenerative medicine.

## Disclosures

None.

## Supplemental Materials

Major Resources Table

Data Supplement Figures I–VII

Data Supplement Movies I–XI

## Supplementary Material


