# Supplementary figures and images for: In Vivo Characterization of Endogenous Cardiovascular Extracellular Vesicles in Larval and Adult Zebrafish
Source: Arterioscler Thromb Vasc Biol. 2021 Jul 15;41(9):2454–68. doi: 10.1161/ATVBAHA.121.316539 (PMC8384253; doi:10.1161/ATVBAHA.121.316539)

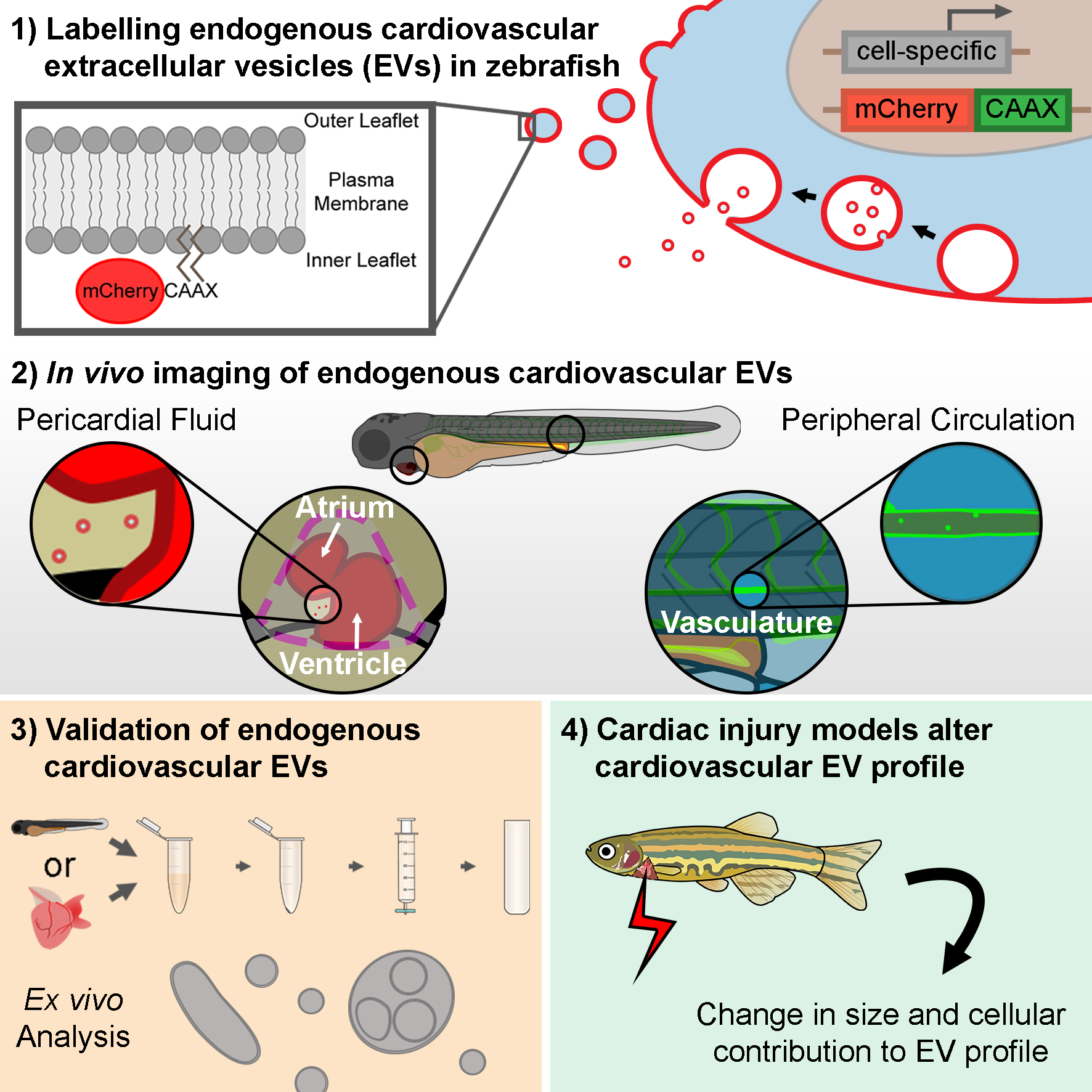

Supplement: Supplementary file 1 [file atv-41-2454-s001.jpg]
