# Supplementary material for: In Vivo Characterization of Endogenous Cardiovascular Extracellular Vesicles in Larval and Adult Zebrafish
Source: Arterioscler Thromb Vasc Biol. 2021 Jul 15;41(9):2454–68. doi: 10.1161/ATVBAHA.121.316539 (PMC8384253; doi:10.1161/ATVBAHA.121.316539)
Supplement: Supplementary file 2 [file atv-41-2454-s002.pdf]

SUPPLEMENTAL MATERIAL

In vivo characterisation of endogenous cardiovascular extracellular vesicles in larval and adult zebrafish

Aaron Scott[1], Lorena Sueiro Ballesteros[2], Marston Bradshaw[1], Chisato Tsuji[1], Ann Power[3], James Lorriman[1], John Love[3], Danielle Paul[1], Andrew Herman[2], Costanza Emanueli[4] and Rebecca J. Richardson[1]*

1 School of Physiology, Pharmacology & Neuroscience, Faculty of Biomedical Sciences, University of Bristol, Bristol, UK

2 Flow Cytometry Facility, Faculty of Biomedical Sciences, University of Bristol, Bristol, UK

3 BioEconomy Centre, The Henry Wellcome building for Biocatalysis, Biosciences, University of Exeter, Exeter, UK.

4 National Heart and Lung Institute, Imperial College London, London, UK; Bristol Heart Institute, School of Clinical Science, University of Bristol, Bristol, UK.

* = corresponding author

Major Resources Table

Genetically Modified Animals (in vivo studies)

Species	Vendor or Source	Background Strain	Sex	Persistent ID / URL
Danio rerio	DOI: 10.1002/dvdy.20252	Tg(*actb2:HRAS-EGFP*)	Male and Female (M/F)	ZDB-ALT-061107-2
Danio rerio	DOI: 10.1096/fj.10-161018	Tg(*tbp:GAL4*); Tg(*UAS:secA5-YFP*)	M/F	ZDB-ALT-110603-3
Danio rerio	DOI: 10.1242/dev.058776	Tg(*kdrl:mCherry-CAAX*)	M/F	ZDB-ALT-110429-3
Danio rerio	DOI: 10.1038/nchembio732	Tg(*myl7:GFP*)	M/F	ZDB-ALT-060719-2
Danio rerio	DOI: 10.1016/j.ydbio.2011.12.006	Tg(*myl7:HRAS-mCherry*)	M/F	ZDB-ALT-120611-2
Danio rerio	DOI: 10.1006/dbio.2002.0711	Tg(*fli1:EGFP*)	M/F	ZDB-ALT-011017-8
Danio rerio	DOI: 10.1182/blood-2010-10-314120	Tg(*mpeg1:EGFP*)gl22	M/F	ZDB-ALT-120117-1

Antibodies

Target antigen	Vendor or Source	Catalog #	Working concentration	Persistent ID / URL
ALIX	Sigma Aldrich	SAB4200476	1:500	https://www.sigmaaldrich.com/catalog/product/sigma/sab4200476
CD63	Santa Cruz Biotechnology	sc-15363	1:200	https://www.scbt.com/p/cd63-antibody-h-193
GAPDH	proteintech	60004-1-IG	1:5000	https://www.ptglab.com/products/GAPDH-Antibody-60004-1-Ig.htm
GFP	proteintech	66002-1-IG	1:2000	https://www.ptglab.com/products/eGFP-Antibody-66002-1-Ig.htm
RFP	MBL International Corporation	PM005	1:1000	https://www.mblintl.com/products/pm005/
SYNTENIN	ThermoFisher Scientific	PA5-42592	1:800	https://www.thermofisher.com/antibody/product/Syntenin-1-Antibody-Polyclonal/PA5-42592
Goat anti-Rabbit IgG (H+L) Cross-Adsorbed Secondary Antibody, HRP	ThermoFisher Scientific	A16104	1:10000	https://www.thermofisher.com/antibody/product/Goat-anti-Rabbit-IgG-H-L-Cross-Adsorbed-Secondary-Antibody-Polyclonal/A16104

Donkey anti-Mouse IgG (H+L), Cross-Adsorbed Secondary Antibody, HRP	biotium	20404	1:10000	https://biotium.com/product/donkey-anti-mouse-igg-hl-highly-cross-adsorbed/?attribute_pa_conjugation=hrp

Other

**Description**	**Source / Repository**	**Persistent ID / URL**
software, Fiji	https://imagej.net/Fiji	Fiji, RRID:SCR_002285
software, Prism7	https://www.graphpad.com/scientific-software/prism/	Prism, RRID:SCR_002798
Software, Imaris	https://imaris.oxinst.com/	Imaris, RRID:SCR_007370
Software, Flowjo	https://www.flowjo.com/	FlowJo, RRID:SCR_008520

Supplemental Figures

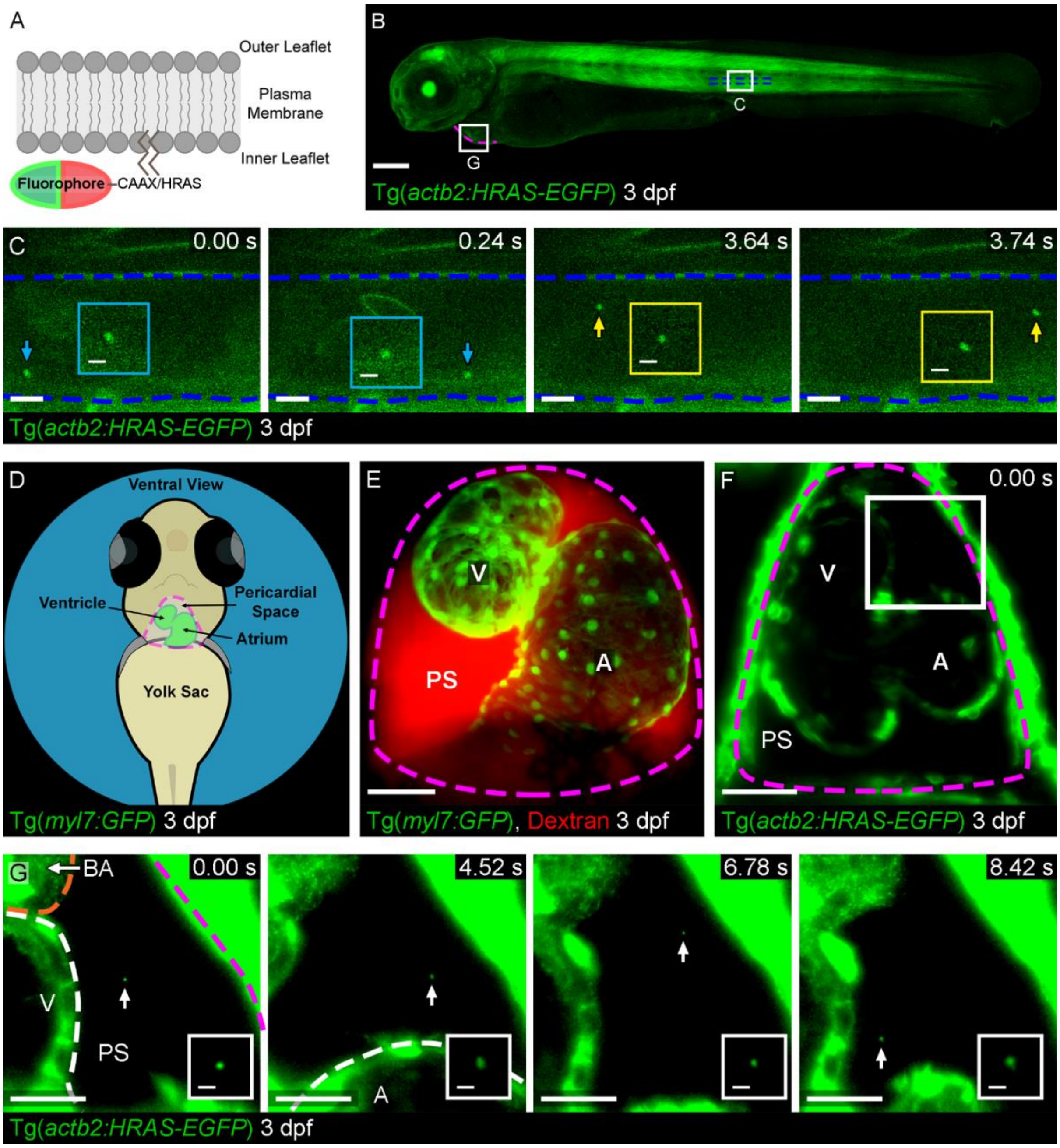

Supplemental Figure I - EV labelling strategy and live imaging in the peripheral circulation and pericardial space. (**A**) Schematic representation of the cell/EV labelling strategy. The fluorophore is tethered to the inner leaflet of the plasma membrane via a CAAX or HRAS motif. (**B**) Overview image of a Tg(*actb2:HRAS-EGFP*) larval zebrafish at 3 dpf. The boxed areas define the regions shown in the image sequences in C and G, as indicated. (**C**) Image sequence of GFP labelled EVs (arrowed) moving through the DA. Blue dashed lines demark the endothelium lining the vessel. Insets show higher magnification views of the arrowed EV. (**D**) Schematic showing the position of the larval heart and pericardial space in a ventral view of a 3 dpf fish. (**E**) Image of the heart in ventral view with fluorescently labelled cardiomyocytes of a Tg(*myl7:GFP*) fish (green) and injected Dextran to demonstrate the extent of the pericardial space (red) at 3 dpf. (**F**) Overview image of the

heart of a Tg(*actb2:HRAS-EGFP*) larval zebrafish at 3 dpf. (**G**) Image sequence of the boxed region in F showing a GFP labelled EV (arrowed and inset) moving through the pericardial space. The magenta dashed line in B,D-G demarks the outer pericardial wall. The orange dashed line in G demarks the bulbus arteriosus. The white dashed line in G demarks the ventricle and atrium. Anterior is to the left in B,C. BA = bulbus arteriosus, V = ventricle, A = Atrium, PS = Pericardial space. Scale bars: B = 200 µm; C = 5 µm; insets in C,G = 2 µm; E,F = 50 µm; G = 20 µm.

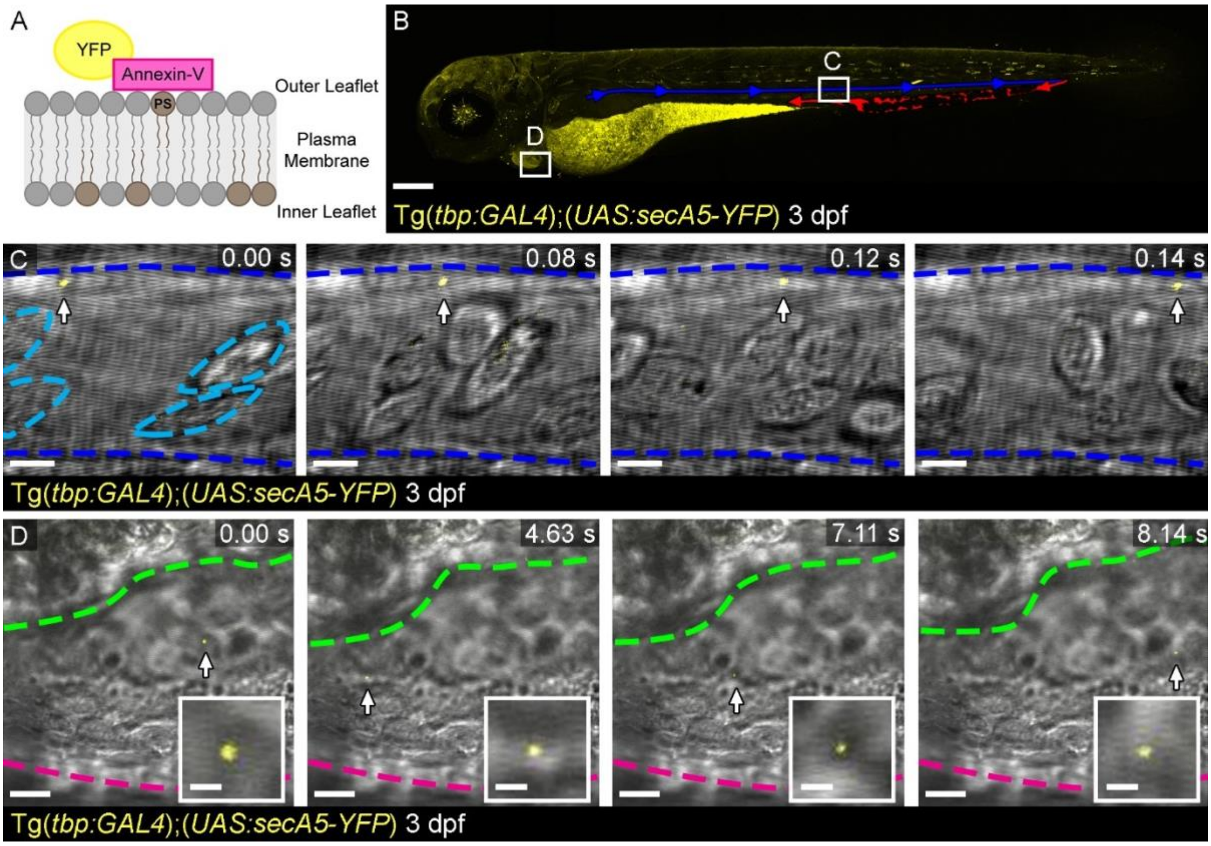

Supplemental Figure II – Live imaging of a global EV labelling strategy via secreted Annexin-V. (**A**) Schematic representation of the Annexin-V labelling strategy. (**B**) Overview image of a larval Tg(*tbp:GAL4*);(*UAS:secA5-YFP*) zebrafish at 3 dpf. Blue indicates the DA and red the caudal haematopoietic (venous) tissue (CHT). Boxes depict the approximate position of the image sequences in C and D. (**C**) Image sequence of an Annexin-V labelled EV moving through the DA. Blood cells can be clearly seen in brightfield. (**D**) Image sequence of an Annexin-V labelled EV (also inset) moving through the pericardial space. The light blue dashed lines in C demark blood cells. The magenta dashed line in D demarks the outer wall of the pericardial space, green outlines the ventricle. Anterior is to the left. Scale bars: B = 200 µm; C = 5 µm; D = 10 µm; insets in D = 2 µm.

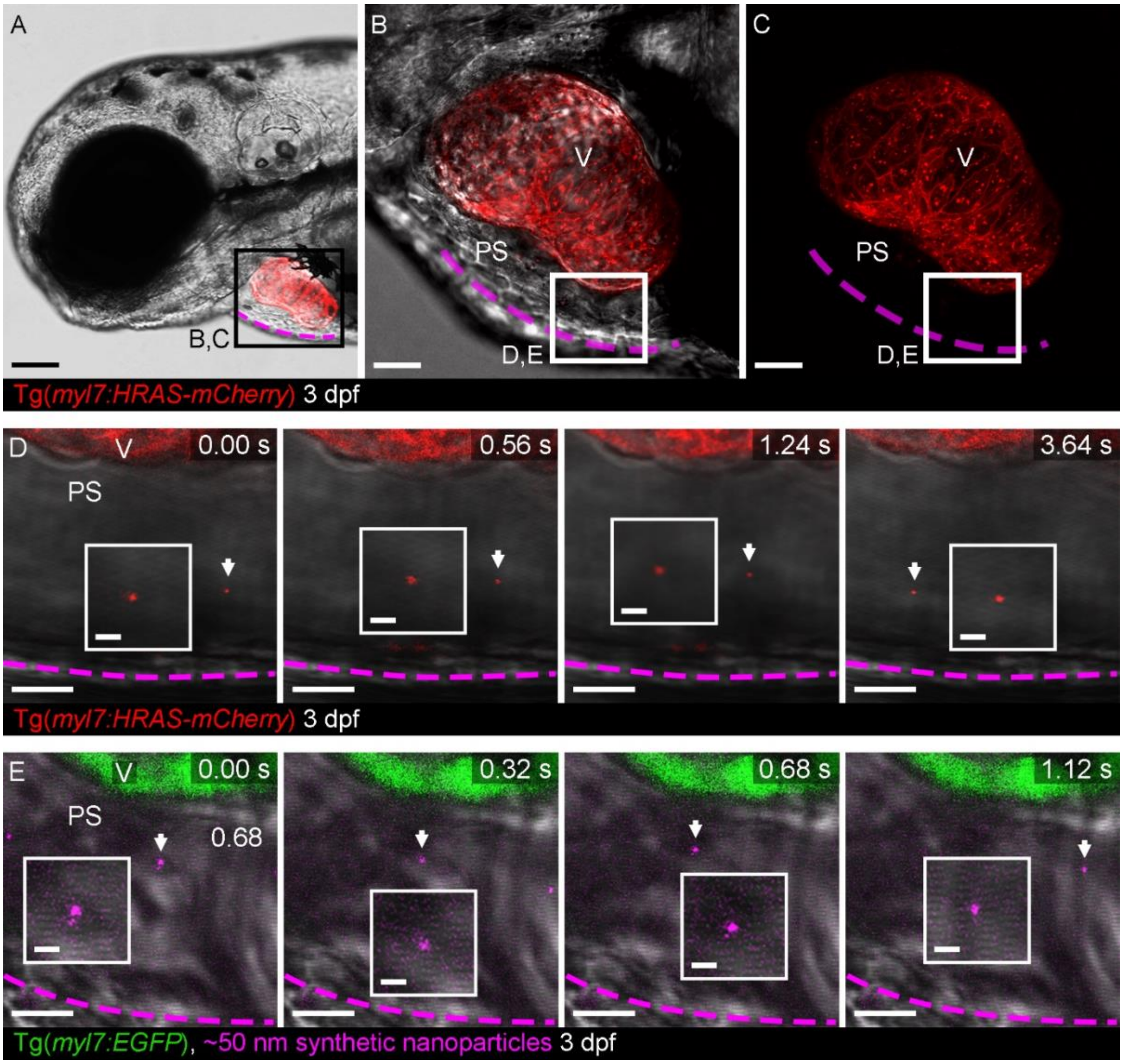

Supplemental Figure III - Live imaging cell-type specific EVs and 50 nm synthetic nanoparticles in the pericardial space. (**A**) Lateral anterior view of a Tg(*myl7:HRAS-mCherry*) larval zebrafish at 3 dpf. The boxed area defines the approximate region shown in B,C. (**B,C**) Optical zoom of the heart shows the entire ventricle; with brightfield (B) and without (C). Boxed area defines the approximate regions shown in D,E. (**D**) Image sequence of higher magnification views of the ventral pericardial space. mCherry+ CM-EVs are observed moving freely through the pericardial space at a distance from the pericardial wall (arrowed and inset). (**E**) Image sequence of higher magnification views of the ventral pericardial space of a Tg(*myl7:GFP*) larvae at 3 dpf. Cy5+ ~50 nm synthetic nanoparticles are observed moving freely through the pericardial space as observed for CM-EVs (arrowed and inset). The magenta dashed line in A-E demarks the outer pericardial wall. Anterior is to the left. V = ventricle, PS = Pericardial space. Scale bars: A = 100 µm; B,C = 25 µm; D,E = 10 µm; insets in D,E = 2 µm.

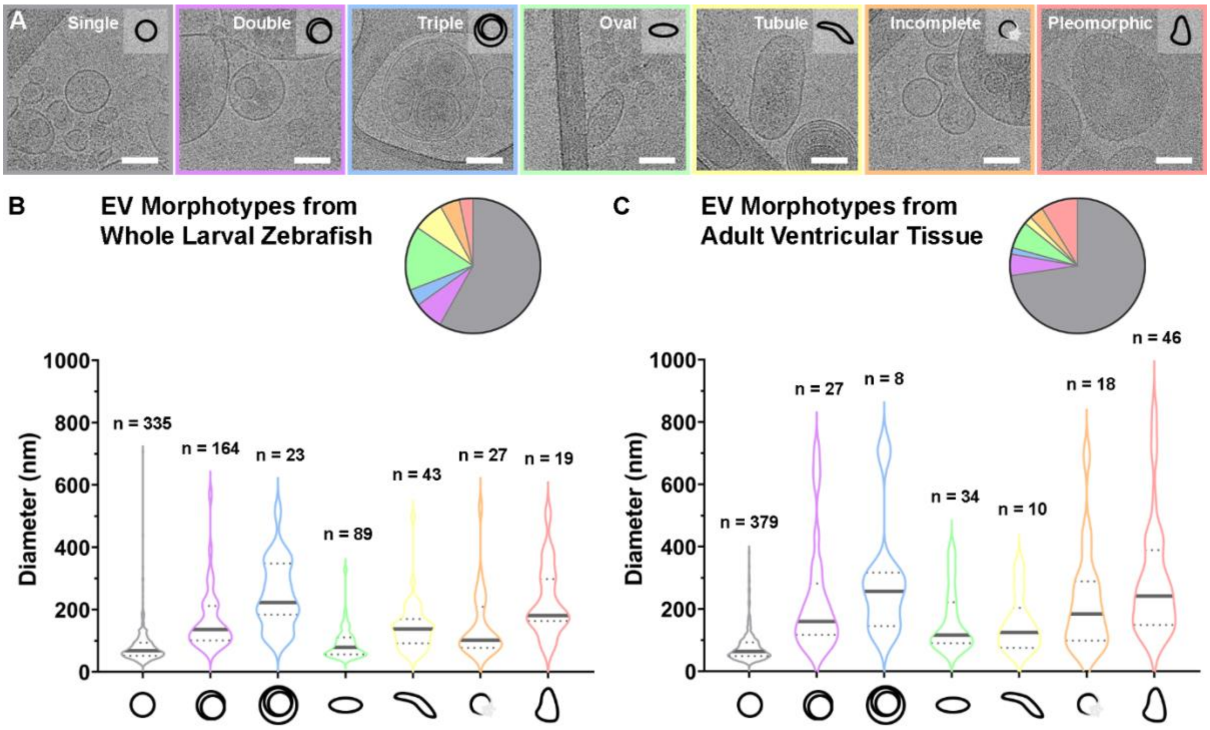

Supplemental Figure IV - Validation of endogenous cardiovascular EVs from larval zebrafish and adult zebrafish cardiac tissue by cryo-EM reveals morphological heterogeneity. (**A**) A panel of representative cryo-EM micrographs showing the different morphotypes quantified in B,C. (**B,C**) Violin plot of the size distribution of different EV morphotypes visualised by cryo-EM. Median and quartile values are shown by the solid and dotted grey lines, respectively. Circle chart shows the proportion of EV morphotypes in the sample. EV samples isolated from whole larval zebrafish (B) and adult zebrafish ventricular tissue (C). Scale bars: A = 100 nm.

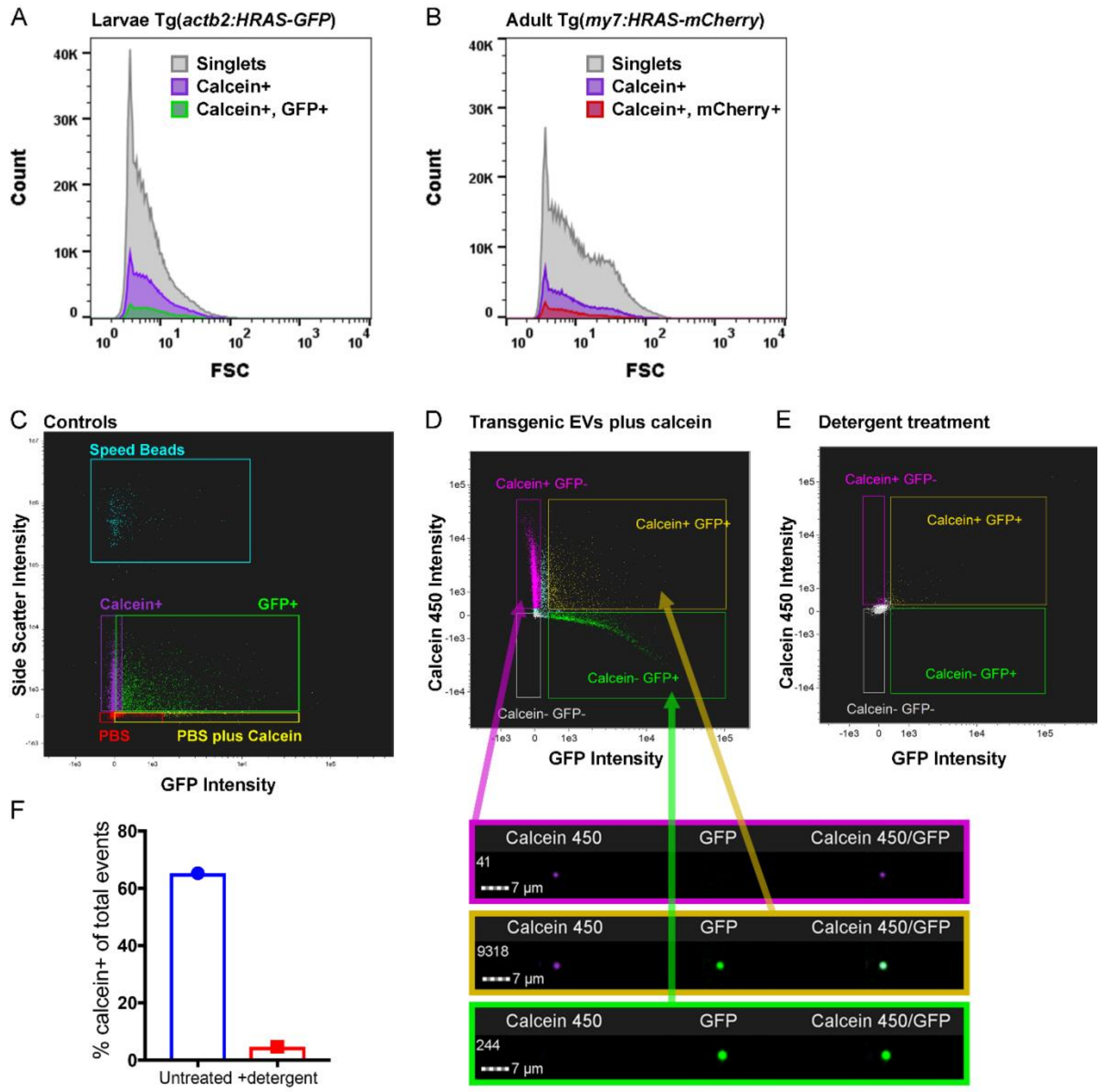

Supplemental Figure V – Fluorescent gating strategy and ImageStream analysis of larval zebrafish EVs. (**A,B**) Analysis of the forward scatter (FSC) profile for all events passing through the singlets gate, those that are calcein+ and those that are calcein+ and positive for cell type specific fluorescence. EVs isolated either from Tg(*actb2:HRAS-GFP*) larvae (A) or Tg(*myl7:HRAS-mCherry*) adult ventricles (B). (**C-F**) ImageStream analysis and quantification of EVs from a pool of 30 whole Tg(*actb2:HRAS-EGFP*) larvae. Control experiments using PBS, PBS plus calcein AM, *actb2*(GFP+) EVs with and without calcein AM and speed beads (1.5 µm diameter, carboxylated polystyrene microspheres, see methods for details) were used to set correct gates for double positive (fluorescence and calcein) EVs (C). Analysis of EVs from Tg(*actb2:HRAS-EGFP*) fish demonstrates different populations of EVs which can be individually visualised (lower panels) (D). Detergent treatment destroys EV integrity further confirming their lipid nature (E,F).

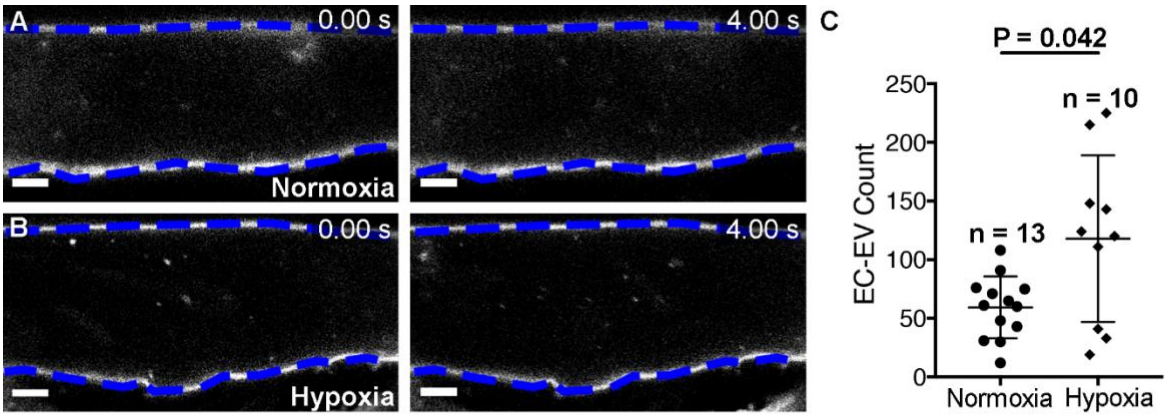

Supplemental Figure VI - EV response to hypoxia in larval zebrafish. (**A,B**) Image sequences of the DA of a 4 dpf Tg(*kdrl:mCherry-CAAX*) fish under normoxic (A) or hypoxic (5% oxygen) conditions (B). (**C**) Quantification of the number of EC-EVs in the DA under normoxic or hypoxic conditions. Statistical analysis in C: Two-tailed Mann-Whitney test. Scale bars: A,B = 5 µm.

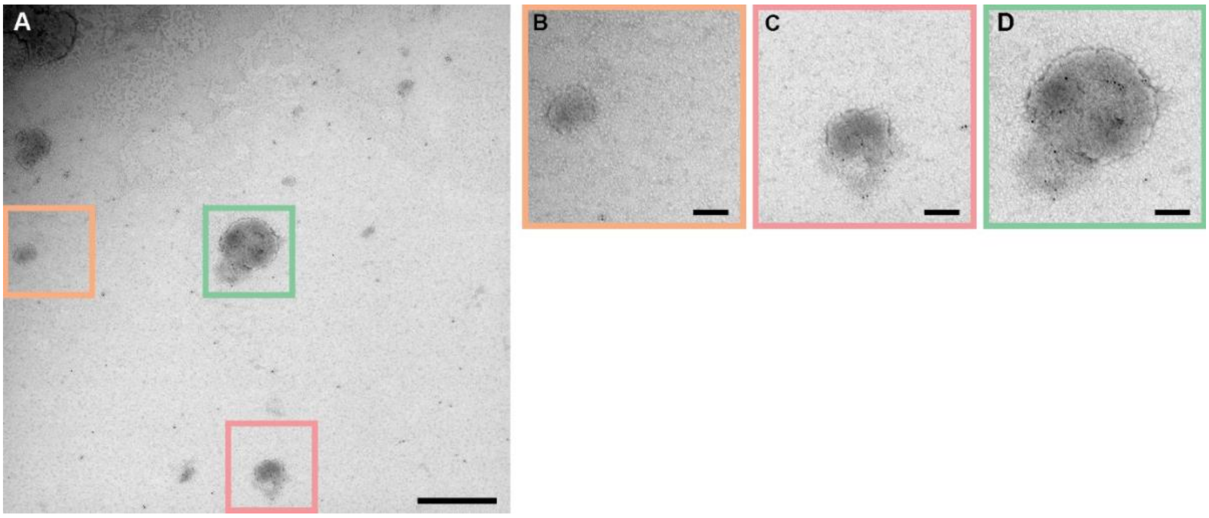

Supplemental Figure VII - Validation of endogenous cardiovascular EVs from adult zebrafish cardiac tissue by immunogold labelling. (A) TEM micrograph of an isolated EV fraction from a pool of Tg(*myl7:HRAS-mCherry*) adult ventricles (n = 30) immunogold labelled with anti-RFP. (**B-D**) A panel of higher magnification views of the boxed regions in A. B shows an example of an unlabelled particle. C,D show examples of gold labelled particles. Scale bars: A = 500 nm; B-D = 100 nm.

Supplemental Videos

Supplemental Video I. Live imaging of *actb2*+ EVs in the peripheral circulation of a larval zebrafish. Image sequence (751 frames = 15 seconds) of *actb2*+ EVs passing through the DA of a 3 dpf Tg(*actb2:HRAS-EGFP*) larval zebrafish. Scale bar = 5 µm.

Supplemental Video II. Live imaging of *actb2*+ EVs in the pericardial space of a larval zebrafish. Image sequence (191 frames = 3.28 seconds) of *actb2*+ EVs travelling through the pericardial fluid of a 3 dpf Tg(*actb2:HRAS-EGFP*) larval zebrafish. Scale bar = 50 µm, 5 µm.

Supplemental Video III. Live imaging of Annexin-V labelled EVs in the pericardial space and peripheral circulation of larval zebrafish. Image sequence (13 frames = 0.26 seconds) of *YFP*+ EV travelling through the DA of a 3 dpf Tg(*tbp:GAL4*);(*UAS:secA5-YFP*) larval zebrafish. Image sequence (327 frames = 13.08 seconds) of *YFP*+ EVs travelling through the pericardial fluid of a 3 dpf Tg(*tbp:GAL4*);(*UAS:secA5-YFP*) zebrafish. Scale bar = 5 µm, 10 µm.

Supplemental Video IV. Live imaging of EC-EVs in the peripheral circulation of a larval zebrafish. Image sequence of mCherry+ EC-EVs passing through the DA (751 frames = 15 seconds) and the CHT (751 frames = 15 seconds) of a 3 dpf Tg(*kdrl:mCherry-CAAX*) larval zebrafish. Scale bar = 5 µm.

Supplemental Video V. Live imaging of an intravascular macrophage in the peripheral circulation of a larval zebrafish. Image sequence (451 frames = 9 seconds) of a GFP+ macrophage containing mCherry+ EC-EVs in the CHT of a 3 dpf Tg(*kdrl:mCherry-CAAX*); Tg(*mpeg1:EGFP*) larval zebrafish. Note the protrusion into the peripheral circulation. Scale bar = 10 µm.

Supplemental Video VI. Live imaging of CM-EVs in the pericardial space of a larval zebrafish. Image sequence (669 frames = 12 seconds) of mCherry+ CM-EVs travelling through the pericardial fluid of a 3 dpf Tg(*myl7:HRAS-mCherry*) larval zebrafish. Scale bar = 50 µm, 5 µm.

Supplemental Video VII. Z-stack of the pericardial space of a Tg(*myl7:GFP*) larval zebrafish injected with dextran. Slice-by-slice view (interval = 0.99 µm, total z-depth = 167.31 µm) of the pericardial space of a Tg(*myl7:GFP*) 3 dpf larvae injected with dextran. Limited cells are seen within the pericardial space. Scale bar = 50 µm.

Supplemental Video VIII. Live imaging CM-EVs and 100 nm synthetic nanoparticles in the pericardial space of a larval zebrafish. Image sequence of mCherry+ CM-EV (260 frames = 5.2 seconds) and 100 nm synthetic nanoparticles (48 frames = 12.96 seconds) travelling through the pericardial space of a 3 dpf Tg(*myl7:HRAS-mCherry*) and Tg(*myl7:GFP*) larval zebrafish respectively. Scale bar = 10 µm.

Supplemental Video IX. Live imaging sequence of EC-EVs in the peripheral circulation of larval zebrafish hypoxia-induced injury models. Image sequences (522 frames = 10.44 seconds) of EC-EVs passing through the DA of 4 dpf Tg(*kdrl:mCherry-CAAX*) larval zebrafish under normoxic and hypoxic conditions (18 hours at 5% Oxygen). Scale bar = 5 µm.

Supplemental Video X. Live imaging sequence of EC-EVs in the peripheral circulation of adult zebrafish. Image sequences (837 frames = 16.62 seconds) of mCherry+ EC-EVs passing through a superficial blood vessel near the gills of a 12-month Tg(*actb2:HRAS-EGFP*); Tg(*kdrl:mCherry-CAAX*) adult zebrafish. Scale bar = 5 µm.

Supplemental Video XI. 3D reconstruction and rendering of the ventricular tissue of a Tg(*kdrl:mCherry-CAAX*); Tg(*mpeg1:EGFP*) adult zebrafish. 3D view of mCherry+ EC-EVs within cardiac macrophages of a Tg(*kdrl:mCherry-CAAX*); Tg(*mpeg1:EGFP*) adult zebrafish heart. Images were reconstructed and rendered using Imaris software. Scale bar = variable with zoom, see video.
